# Phenotypic Anchoring of Gene Expression Changes during Estrogen-Induced Uterine Growth

**DOI:** 10.1289/txg.7345

**Published:** 2004-10-07

**Authors:** Jonathan G. Moggs, Helen Tinwell, Tracey Spurway, Hur-Song Chang, Ian Pate, Fei Ling Lim, David J. Moore, Anthony Soames, Ruth Stuckey, Richard Currie, Tong Zhu, Ian Kimber, John Ashby, George Orphanides

**Affiliations:** ^1^Syngenta Central Toxicology Laboratory, Alderley Park, Cheshire, United Kingdom; ^2^Syngenta Biotechnology Inc., Research Triangle Park, North Carolina, USA

**Keywords:** estrogen, gene expression, microarray, phenotypic anchoring, uterus

## Abstract

A major challenge in the emerging field of toxicogenomics is to define the relationships between chemically induced changes in gene expression and alterations in conventional toxicologic parameters such as clinical chemistry and histopathology. We have explored these relationships in detail using the rodent uterotrophic assay as a model system. Gene expression levels, uterine weights, and histologic parameters were analyzed 1, 2, 4, 8, 24, 48, and 72 hr after exposure to the reference physiologic estrogen 17β-estradiol (E_2_). A multistep analysis method, involving unsupervised hierarchical clustering followed by supervised gene ontology–driven clustering, was used to define the transcriptional program associated with E_2_-induced uterine growth and to identify groups of genes that may drive specific histologic changes in the uterus. This revealed that uterine growth and maturation are preceded and accompanied by a complex, multistage molecular program. The program begins with the induction of genes involved in transcriptional regulation and signal transduction and is followed, sequentially, by the regulation of genes involved in protein biosynthesis, cell proliferation, and epithelial cell differentiation. Furthermore, we have identified genes with common molecular functions that may drive fluid uptake, coordinated cell division, and remodeling of luminal epithelial cells. These data define the mechanism by which an estrogen induces organ growth and tissue maturation, and demonstrate that comparison of temporal changes in gene expression and conventional toxicology end points can facilitate the phenotypic anchoring of toxicogenomic data.

Gene expression profiling, used within the existing framework of toxicologic assessment, promises to advance significantly the mechanistic understanding and prediction of adverse effects. To benefit fully from the opportunities offered by gene expression profiling, we must first understand the relationships between changes in gene expression and alterations in traditional toxicology parameters. The process by which gene expression changes are linked to changes in phenotype has been termed “phenotypic anchoring” (Cunningham et al. 2003; Paules 2003; Schmidt 2003). This approach has been used successfully to identify groups of genes whose expression correlates with specific pathologic changes during griseofulvin-induced chronic liver injury (Gant et al. 2003), renal toxicity (Amin et al. 2004), furan-mediated hepatotoxicity (Hamadeh et al. 2004), and aceta-minophen-induced hepatotoxicity (Heinloth et al. 2004). In the present study we used phenotypic anchoring, in conjunction with gene ontology analysis, to define the transcriptional program associated with the response of the rodent uterus to a reference estrogen and to identify groups of genes that may drive specific histologic changes.

The immature mouse uterus is a major estrogen-responsive organ and forms the basis for a reference assay of estrogenic activity of chemicals (Owens and Ashby 2002). The physiologic response of the uterus to exogenous estrogens has been documented in detail (Clark and Mani 1994). The immature mouse uterus is sensitive to elevations in endogenous levels of 17β -estradiol (E_2_) that occur during puberty. E_2_ releases the immature uterus from quiescence and promotes cell proliferation and differentiation. The initial effects of E_2_ are rapid (4–6 hr) and involve the uptake of fluid resulting from hyperemia and vasodilation of uterine capillaries, which causes the uterus to swell. This phenomenon is termed “water imbibition” and increases the availability of substrates and ions required for growth. Another early event is an increase in overall levels of mRNA and protein synthesis. The uterus then enters a proliferative phase that is responsible, at least in part, for the large increase in uterine weight that occurs 16–30 hr after E_2_ exposure. Later responses mimic the changes in uterine physiology that accompany the onset of puberty and include alterations in the surface of the luminal epithelia.

Although the events described above have been characterized at the physiologic level, little is known about how E_2_, acting through the estrogen receptors ER-αand ER-β, coordinates at the molecular level the myriad cellular processes involved, despite significant progress in elucidating the molecular mechanisms by which ERs regulate gene expression *in vitro* (Hall et al. 2001; McKenna and O’Malley 2002; Metivier et al. 2003; Moggs and Orphanides 2001; Moggs et al. 2003; Tremblay and Giguere 2002). Our data reveal the transcriptional program associated with E_2_-induced uterine growth. We show that E_2_ induces a tightly coordinated transcriptional program that regulates successive and interlinked cellular processes during the uterotrophic response. Moreover, by comparing changes in gene expression with alterations in uterine weight and histology, we have identified classes of genes that may drive specific histologic changes in the uterus, including fluid uptake, coordinated cell division, and remodeling of the luminal epithelial cell layer in preparation for embryo implantation. Our data also provide novel insights into how E_2_ initiates paracrine signaling events, recruits immune and inflammatory cells, increases mRNA and protein synthesis, and suppresses apoptosis.

These data describe, at an unprecedented level of detail, how E_2_ induces organ growth and maturation and provide a paradigm for understanding the mechanisms of action of other nuclear receptors. Furthermore, this study demonstrates that analysis of the temporal associations between a chemically induced transcriptional program and the accompanying histologic changes can provide valuable insight into the relationships between gene expression changes and phenotypic alterations.

## Materials and Methods

### Animals

Female Alpk:Ap_f_CD-1 mice (19–20 days old), weighing no more than 14 g on arrival in the laboratory, were obtained from a barriered animal breeding unit (AstraZeneca, Macclesfield, Cheshire, UK). The animals were housed five per cage in solid-bottom cages and allowed to acclimatize for 24 hr. They were allowed RM1 diet (Rat and Mouse No. 1; Special Diet Services Ltd., Witham, Essex, UK) and water ad libitum for the duration of the study. All animal experimentation described in this article was conducted in accord with accepted standards (local and national regulations) of humane animal care. Group sizes of 10 animals were used for the first two of the three replicate studies. Five animals per group were used in the third replicate study.

### Uterotrophic Assays

The mice were given a single subcutaneous injection of E_2_ (400 μg/kg) or arachis oil (AO; vehicle control) using a dosing volume of 5 mL/kg body weight. A single dose of E_2_ was used to avoid the complex transcriptional program that may result from the standard uterotrophic assay exposure regime (i.e., repeated administration on 3 consecutive days; Odum et al. 1997). The relatively high dose level of 400 μg/kg was chosen to ensure a sustained and significant increase in blotted uterine weight during the 72-hr sampling period (Supplemental Data, Figure 1). No overt toxicity was observed during the 72-hr exposure to E_2_ (400 μg/kg). All animals were terminated at the appropriate time using an overdose of halothane (Concord Pharmaceuticals Ltd., Essex, UK) followed by cervical dislocation. Vaginal opening was recorded, and the uterus was then removed, trimmed free of fat, gently blotted, and weighed. Blotted uterine weights were analyzed by covariance with terminal body weights (SAS Institute Inc. 1999). Half of each left uterine horn was fixed in 10% formol saline and processed to paraffin wax for histologic analysis (Odum et al. 1997). The mean thickness of the endometrial and epithelial cell layers, indicators of cellular hypertrophy, were calculated based on the assessment of 10 locations on hemotoxylin- and eosin-stained longitudinal uterine sections for each animal. All hypertrophy data were assessed for statistical significance by analysis of variance (ANOVA). The remainder of the uterus was snap frozen in liquid nitrogen and stored at −70°C for RNA extraction.

### Mitotic Index

The total number of mitotic figures in each uterus section was counted, noting the tissue location, and the area of the section was measured using a KS400 image analysis system (Imaging Associates, Bicester, UK). The number of mitotic figures per square millimeter was calculated, and the frequency after administration of E_2_ was compared with the frequency seen after the administration of AO using an appropriate statistical procedure. The number of mitoses per square millimeter was considered by a fixed-effects ANOVA allowing for treatment, time, and the treatment by time interaction. Analyses were carried out using the MIXED procedure in SAS, version 8.2 (SAS Institute Inc. 1999). Contrasts within the treatment by time interaction provided estimates of differences in E_2_ and control response at each time point. These were compared statistically using a two-sided Student *t*-test based on the error mean square in the ANOVA.

### Transcript Profiling and Data Analysis

Three independent biologic replicates of the entire time course study for E_2_-treated and time-matched AO-treated groups of animals were used to generate transcript profiling data and for subsequent statistical analysis. To minimize the effect of any interanimal variability, total RNA was isolated from the pooled uteri for each treatment group (*n* = 10 in the first two studies; reduced to *n* = 5 for the last study because of highly similar transcriptional responses being obtained in replicate studies 1 and 2) using RNeasy Midi kits (Qiagen Ltd., Crawley, West Sussex, UK). Biotin-labeled complementary RNAs were synthesized using the Enzo Bioarray HighYield RNA transcript labeling kit and hybridized to Affymetrix murine U74-Av2 GeneChips as described previously (Zhu et al. 2001) and in the Affymetrix GeneChip expression analysis technical manual (Affymetrix, Inc. 2002). Probe arrays were scanned and the intensities were averaged using Microarray Analysis Suite 5.0 (Affymetrix, High Wycombe, UK). The mean signal intensity of each array was globally scaled to a target signal value of 500. To select E_2_-responsive genes, each gene was subjected to a mixed-model ANOVA allowing for treatment, time, and the treatment by time interaction as fixed effects and replicate study as a random effect. The use of mixed ANOVA models for the analysis of differential gene expression in microarray experiments has been previously described (Churchill 2004; Cui and Churchill 2003). Analyses were carried out using the MIXED procedure in SAS, version 8.2 (SAS Institute Inc. 1999). Contrasts within the treatment by time interaction provided estimates of differences in E_2_ and control response at each time point. These were compared statistically using a two-sided Student *t*-test based on the error mean square in the ANOVA [Supplemental Data, Table 1 (http://ehp.niehs.nih.gov/txg/members/2004/7345/supplemental.pdf)]. Data for genes exhibiting significant changes in expression (*p* < 0.01, two-sided) at one or more time points were then exported into GeneSpring 6.0 (SiliconGenetics, Redwood City, CA, USA), and a data transformation (values < 0.01 set to 0.01) and per-chip normalization (to the 50th percentile) were applied. Genes that did not have a *Present* detection call (Affymetrix) in any of the 14 treatment groups were removed from further analysis. Ratios of changes in gene expression were then calculated by normalizing each E_2_-treated sample to its corresponding time-matched vehicle (AO)-treated control. GeneChip data sets for the three independent biologic replicates were interpreted in log of ratio analysis mode, with biologic replicates being selected as a noncontinuous parameter. A total of 3,538 E_2_-responsive genes exhibiting a minimum of 1.5-fold up- or down-regulation in at least one time point were then subjected to gene tree–based hierarchical clustering (Pearson correlation). To identify genes that function in specific biologic pathways, these 3,538 genes were further filtered using functional annotations derived from the NetAffx database‚ Analysis Center (Liu et al. 2003; http://www.affymetrix.com/analysis/index.affx), together with manual annotations from published literature, before hierarchical clustering using GeneSpring. Gene names used in this article (see Appendix) were derived by homology searching of nucleotide sequence databases (BLASTn; http://www.ncbi.nih.gov/BLAST/) using Affymetrix probe target sequences and the interrogation of NetAffx (Liu et al. 2003) database. All genes described in the figures and text showed statistically significant alterations in expression in all three replicate studies. MIAME (Minimum Information About a Microarray Experiment)-compliant microarray data for the three independent replicate studies are available as supplementary information and have been submitted to the Gene Expression Omnibus (GEO) database (http://www.ncbi.nlm.nih.gov/geo/).

### Quantitative Real-Time Polymerase Chain Reaction

Uterine RNA was isolated and purified from all E_2_-treated and time-matched vehicle control groups (each consisting of pooled uteri) in all three replicate time course studies using the Qiagen RNeasy Midi kit (Qiagen). Before reverse transcription, RNA was treated with Dnase I (DNA-free kit; Ambion, Huntington, UK) to remove any contaminating genomic DNA. For each pool, 2 μg total RNA was reverse transcribed in a 25-μL reaction using SuperScript II (Invitrogen, Paisley, UK) and oligo-dT primer according to the manufacturer’s instructions. Polymerase chain reaction (PCR; 25 μL) containing 2 μL first-strand cDNA (1:10 dilution), 12.5 μL of SYBR Green PCR Master Mix (Applied Biosystems, Warrington, UK), and 0.3 μM each of forward and reverse primers were run for 40 amplification cycles in an ABI PRISM 7700 Sequence Detection System (Applied Biosystems). Cycling conditions were 50°C for 2 min, 9°C for 10 min, 95°C for 15 sec, and 60°C for 1 min. All reactions were run in triplicate. Real-time (RT) PCR primers for *FOS* (5′-CTGTGGCCTCCCTGGATTTG-3′and 5′-TGAGAAGGGGCAGGGTGAAG-3′), *LTF* (5′-CGGGGGCCTTCAGACCATC-3′and 5′-CTAAAGTGACAGCAGGG AGTG-3′), and the control gene *RPB1* (5′ - GTTCTGGACCCCATTTTTGATAGGC-3′ and 5′-CAGGGGACTGGCAGGGTAACAA-3′) were designed using Primer Express software (version 1.5; Applied Biosystems) to generate amplicons within their corresponding Affymetrix probe set target sequences.

## Results

### Histologic Changes and Increases in Uterine Weight

**Figure 1:**
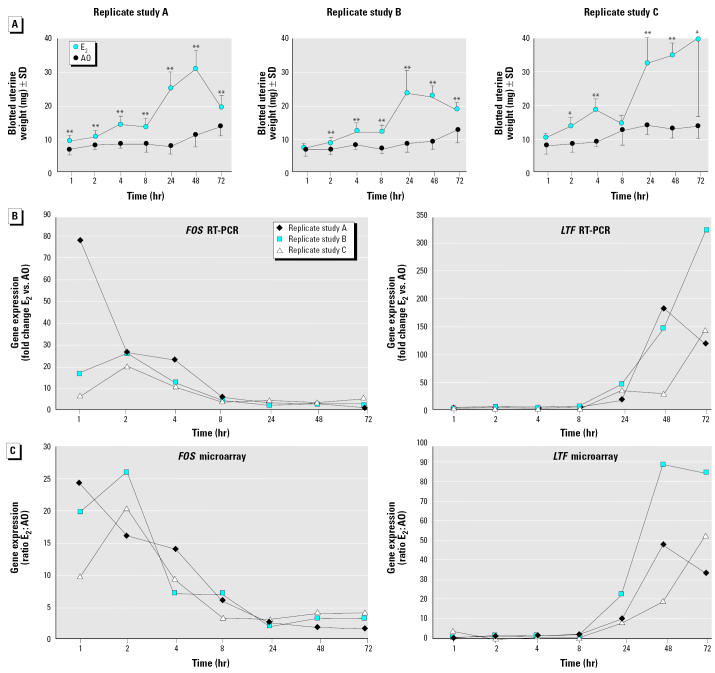
Uterotrophic response to a single dose of E_2_. (*A*) Uterine blotted weight. Data for replicate studies A and B are mean ± SD from 10 immature female mice in each treatment group. Five animals per group were used in replicate study C. (*B*) Temporal expression profile of the estrogen-responsive genes complement component C3 and C-FOS. Quantitative RT-PCR analysis of *FOS* and *LTF* gene expression from three independent time-course studies (*A–C*) and comparison with microarray data. Each RT-PCR data point represents a fold value, obtained using the comparative *C_t_* (threshold cycle) method, for E_2_-induced change in gene expression relative to time-matched vehicle controls. The fold induction value is relative to the endogenous control gene *RPB1* and to treatment, that is, estrogen/untreated. Microarray data are ratios (E_2_:time-matched vehicle control) of normalized Affymetrix GeneChip signal intensities (see Figure 3A and “Materials and Methods”).
*p < 0.05;
**p < 0.01.

Our aim was to identify the genes and molecular networks associated with the uterotrophic response and to define the relationships between gene expression changes and histologic alterations. To this end, we gave immature female mice a single subcutaneous injection of E _2_ (400 μg/kg) or vehicle and used DNA microarrays to measure uterine gene expression profiles at seven different times (1, 2, 4, 8, 24, 48, and 72 hr) after exposure. To facilitate the phenotypic anchoring of expression changes, we also measured blotted uterine weights and determined the average heights of the luminal epithelium and stromal endometrium for each animal. Three independent replicate experiments were carried out to allow a rigorous statistical analysis of the gene expression data (see “Materials and Methods”). We chose to use a single dose of E_2_ to avoid the complex transcriptional program that may result from the standard uterotrophic assay exposure regime in which test compound is dosed by repeated administration on 3 consecutive days (Odum et al. 1997). This dose induced a sustained increase in blotted uterine weight that was similar in the three replicate experiments (Figure 1A). In each replicate experiment, a significant increase (*p* < 0.01) in uterine weight was observed 4 hr after exposure to E_2_ and reached maximal levels between 24 and 72 hr (Figure 1A).

**Figure 2:**
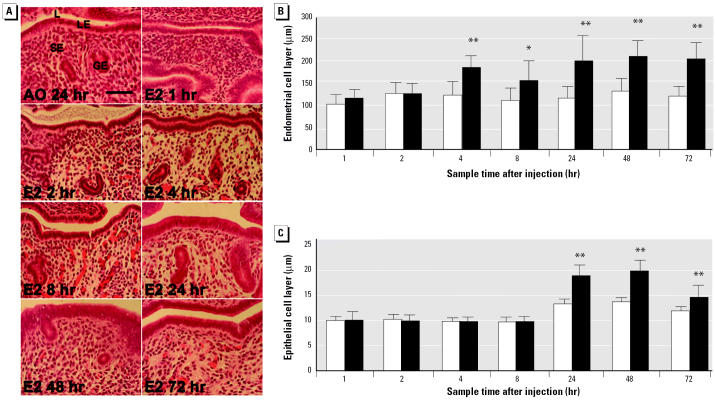
Histologic analysis of uterotrophic response to a single dose of E_2_. (*A)* Panels show longitudinal 0.3-μm–thick paraffin sections of uteri stained with hematoxylin and eosin; bar = 50 μm. Luminal space (L), luminal epithelium (LE), stromal endothelium (SE), and glandular epithelium (GE) are indicated. (*B*) Height of stromal endothelial cell layer. (*C*) Height of luminal epithelial cell layer. Data in *B* and *C* are mean ± SD from 10 immature female mice in each treatment group. Solid bars, E_2_; open bars, AO.
*p < 0.05;
**p < 0.01.

Histologic analysis of uterine sections revealed the cellular changes associated with the increase in uterine weight between 1 and 72 hr (Figure 2A). Consistent with previous reports (Clark and Mani 1994), the weight increase that occurred within 4 hr of exposure (Figure 1A) was associated with thickening of the stromal endometrium (Figure 2B) resulting from the uptake of fluid. The larger increase in uterine weight that occurred between 8 and 24 hr was due to hypertrophy and cell proliferation (Kaye et al. 1971; Quarmby and Korach 1984), which caused an increase in thickness of the luminal epithelium between 8 and 24 hr (Figure 2C). We conclude that the single dose of E _2_ used induced a conventional uterotrophic response. Furthermore, the expression profiles of two classical E_2_-responsive genes, lactotransferrin (*LTF* ; Liu and Teng 1992) and the proto-oncogene *C-FOS* (Weisz and Bresciani 1988), demonstrate that E_2_ elicited a robust transcriptional response that was similar in the three replicate experiments (Figure 1B).

### Multistep Method for Analysis of Gene Expression Changes

**Figure 3:**
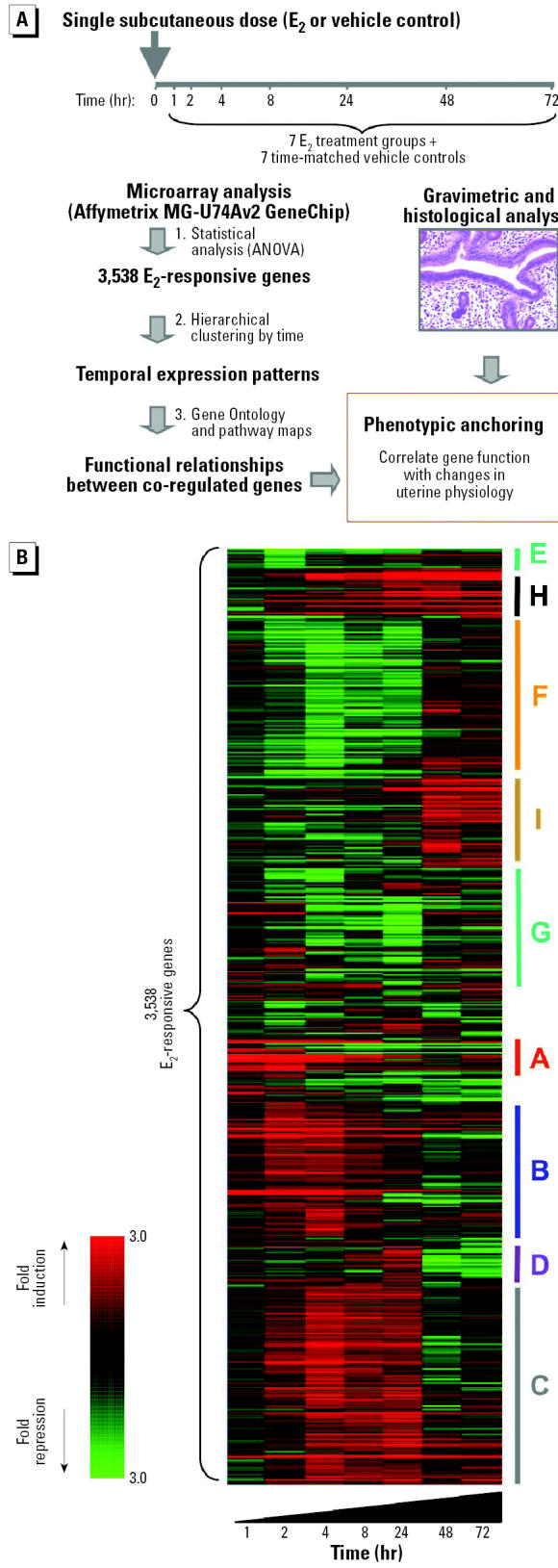
(*A*) Experimental strategy for phenotypic anchoring of E_2_-responsive genes during uterotrophic response. Three independent biologic replicate studies were performed in which we analyzed seven different time points for E_2_-treated animals and the equivalent time points for vehicle-treated animals. (*B*) Staged transcriptional response of the immature mouse uterus to E_2_. Gene tree generated by hierarchical clustering of 3,538 E_2_-responsive genes showing clusters (labeled A–I) of temporally co-regulated genes. The genes clustered in groups A–I are further annotated using gene ontology analyses in Figures 4–7. The color scale indicates the mean fold change of E_2_-induced gene expression relative to time-matched AO-treated control samples (based on the three independent studies shown in Figure 1A).

Uterine RNA from the seven time points for each of the E_2_-treated and time-matched vehicle control groups was analyzed using Affymetrix MG-U74Av2 GeneChips. A total of 42 microarray data sets were collected for the three replicate experiments. We used a multistep method to analyze the microarray gene expression data (Figure 3A). First, data were filtered and subjected to statistical analyses to identify the 3,538 genes with altered expression in E_2_-treated mice (*p* < 0.01 and > 1.5-fold) during at least one time point (see “Materials and Methods”). Unsupervised hierarchical clustering was then used to group these genes into co-regulated clusters (Quackenbush 2002; Figure 3B), revealing a complex multistage transcriptional response to E_2_ in the uterus (gene clusters A–I in Figure 3B). To gain an overview of the predominant molecular functions and biologic pathways that were regulated at the transcriptional level during the uterotrophic response to E_2_, we interrogated the 3,538 E_2_-responsive genes using the GOStat gene ontology mining tool (http://gostat.wehi.edu.au) (Beissbarth and Speed 2004). This approach revealed that E_2_ targets predominantly genes involved in protein metabolism, cell cycle, cell proliferation, DNA replication, RNA metabolism, mRNA transcription, and blood vessel development [Supplemental Data, Table 2 (http://ehp.niehs.nih.gov/txg/members/2004/7345/supplemental.pdf)]. Next, we used a supervised clustering approach using customized gene ontology definitions (see “Materials and Methods”) to identify gene functions that were predominant in each co-regulated cluster in Figure 3B. This revealed that E_2_ regulates each class of gene during a narrow window of time and suggests that E_2_ induces uterine growth and maturation by regulating successively the activities of different biologic pathways (described below). Finally, we analyzed the temporal associations between the gene expression program and alterations in uterine weight and histology to anchor the gene expression changes to alterations in uterine phenotype. These associations are described below.

### Phase 1: Rapid Induction of Transcriptional Regulators and Signaling Components by E_2_

**Figure 4:**
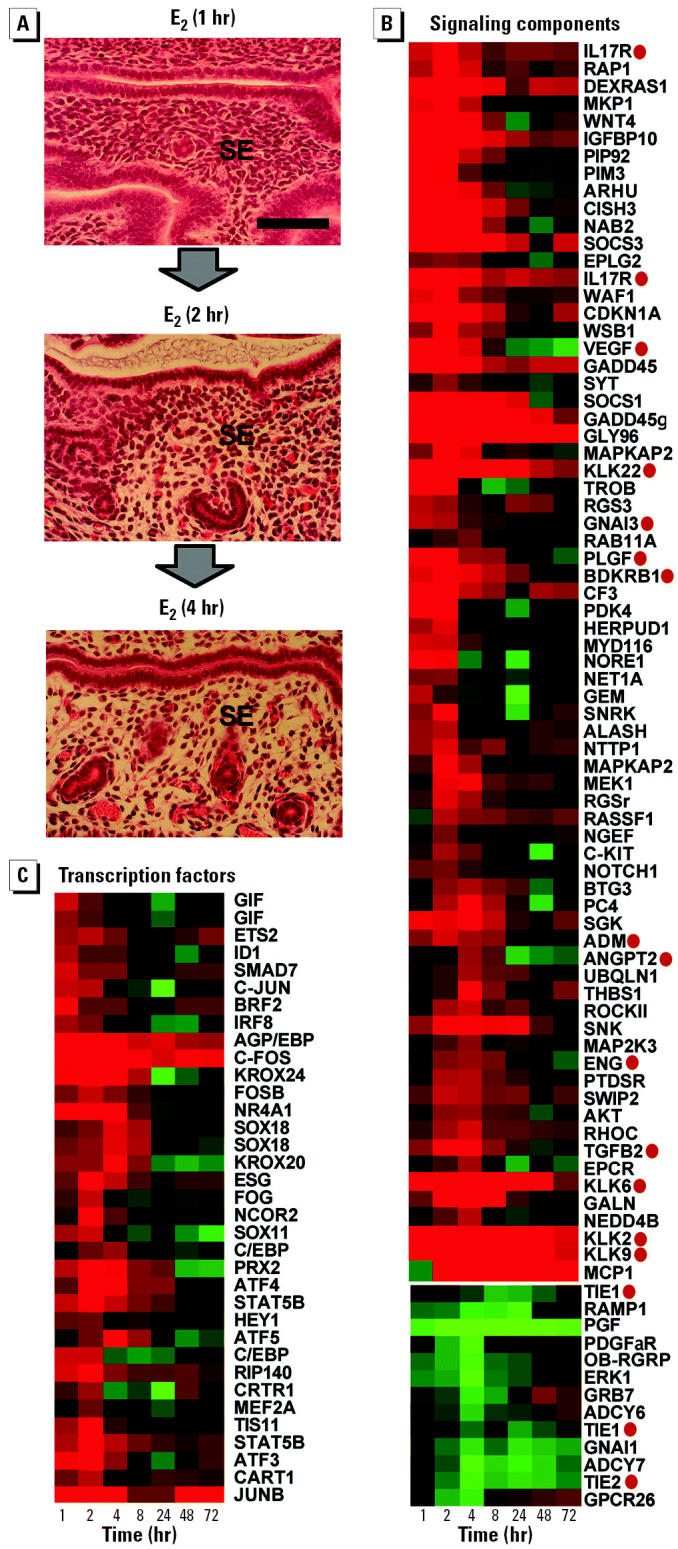
Phase 1: rapid induction of transcriptional regulators and signaling components by E_2_. (*A*) Water imbibition and increased vascular activity in stromal endothelium (SE) 2 and 4 hr after a single dose of E_2_. Longitudinal 0.3-μm–thick paraffin sections of uteri stained with hematoxylin and eosin are shown. Scale bar, 50 μm. (*B*) Coordinate expression of genes encoding signaling components. Genes marked with a red circle have functions associated with altered vascular permeability and may drive the water imbibition seen at this time. (*C*) Coordinate expression of genes encoding transcription factors. Detailed quantitative data for genes encoding AP-1 transcription factors are shown in Figure 8B. Gene trees were generated by supervised hierarchical clustering; genes with related functions were selected from clusters of temporally co-regulated E_2_-responsive genes (Figure 3B) using universal gene ontology descriptions. The color scale for fold change in expression is identical to that used in Figure 3B. Data derived from independent Affymetrix probe sets are shown for *GIF* and *SOX18*. See Appendix for gene nomenclature and Affymetrix probe sets.

The first 4 hr of the uterotrophic response is characterized by the influx into the uterus of fluid that provides the nutrients and ions required for growth (Clark and Mani 1994). This leads to decompaction of stromal cells (Figure 4A) and thickening of the stromal endometrial layer at 4 hr (Figure 2B). This first phase of the uterotrophic response is accompanied by the rapid and transient regulation of genes encoding components of intra- and inter-cellular signaling pathways (Figure 4B) and sequence-specific transcriptional regulators (Figure 4C). Most of these genes show maximal expression between 1 and 4 hr, suggesting that the transcriptional effects of E _2_, mediated via ER- αand ER-β, are amplified rapidly through the induction or modulation of multiple transcriptional and nontranscriptional signaling pathways.

### Signaling Genes

The signaling genes rapidly up-regulated by E_2_ function in a broad array of signal transduction pathways (Figure 4B). These genes include protein kinases (*AKT, MEK1, PIM3*), growth factors (*VEGF*, *PLGF*), GTPases (*RHOC, RAB11A, DEXRAS1*), cytokine signaling proteins (*MCP1, SOCS1, SOCS3*, *WSB1, IL17R*), and a Wnt signaling factor (*WNT4*). Several E _2_ -induced genes may act to attenuate initial signaling events (e.g., the protein phosphatase *MKP1* negatively modulates MAP kinase activity). Strikingly, many of the signaling genes induced within 4 hr of E_2_ exposure have roles in the regulation of vascular permeability in other tissues, suggesting that they may be involved directly in initiating the influx of fluid into the uterus at this time (Figure 4B). These genes include angiogenic/vascular cell growth factors (*VEGF, PLGF, ADM, ANGPT2, TGFB2*), vasoactive serine proteases (*KLK2, KLK6, KLK9, KLK22*), and vascular endothelial receptors (*IL17R, BDKRB1, ENG, GNA13*). Furthermore, the vascular growth factor receptors *TIE1* and *TIE2* are rapidly down-regulated in response to E_2_ (Figure 4B), which may serve to attenuate the uptake of fluid after 4 hr. Collectively, these genes shed light on the mechanism by which E_2_ promotes fluid uptake in the uterus and provide a clear link between gene expression changes and histologic changes occurring at this time.

### Transcriptional Regulators

The sequence-specific transcription factors induced during the first 4 hr of the response can be divided into four main classes (Figure 4C). The first contains members of the Jun, Fos, and ATF subgroups of transcription factors (*C-FOS, FOSB, C-JUN, JUNB, ATF3, ATF4, ATF5*) that form AP-1 dimers implicated in the regulation of cell proliferation and survival (Shaulian and Karin 2001). The second class contains genes that control cell differentiation during the development of a number of tissues (*SOX11, SOX18, HEY1, CART1, PRX2, SMAD7, ID1*). The early induction of members of this class suggests that E_2_ deploys a diverse range of gene expression networks to control cell growth and differentiation in the uterus. The third class contains two genes that encode co-regulators for nuclear receptors (*RIP140*, *NCOR2*), suggesting that these may act to modulate ER-mediated responses to E_2_ in the uterus. The fourth class of genes encodes presumed transcriptional regulators of unknown function (e.g., *GIF*).

We conclude that the initial response to E_2_ serves to *a*) modulate the activities of intra- and intercellular signaling pathways that, among other functions, promote vascular permeability and fluid uptake and *b*) up-regulate the expression levels of transcription factors that promote growth and differentiation. These early gene expression changes facilitate the amplification of the originating hormonal signal and set into motion the series of events that result in uterine growth and differentiation.

### Phase 2: Coordinated Induction of Genes Required for mRNA and Protein Synthesis

**Figure 5:**
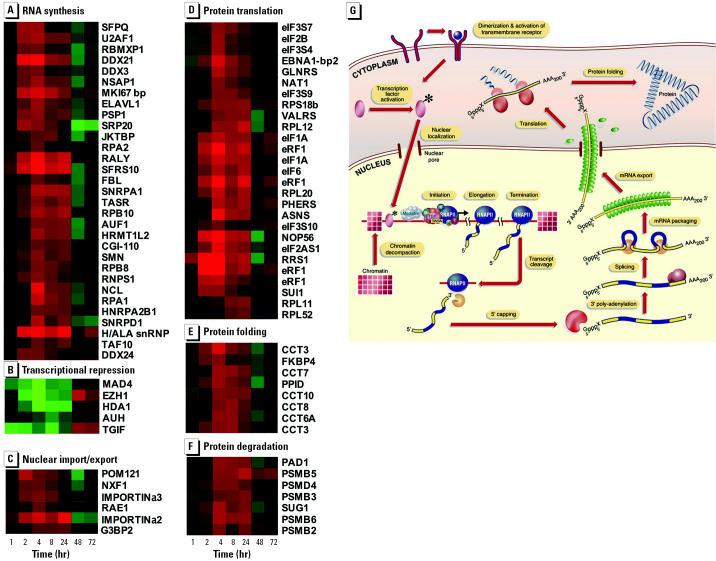
Phase 2: coordinated induction of genes required for mRNA and protein synthesis. Coordinated expression of genes involved in (*A*) RNA synthesis, (*B*) transcriptional repression, (*C*) nuclear import/export, (*D*) protein translation, (*E*) protein folding, and (*F*) protein degradation. Gene trees were generated as described in Figure 4. Data derived from independent Affymetrix probe sets are shown for *eIF1A*, *eRF1*, and *CCT3*. See Appendix for gene nomenclature and Affymetrix probe sets. (*G*) Schematic overview of RNA and protein synthesis in eukaryotes, showing machinery involved in each step of the process. Reprinted from Orphanides and Reinberg (2002), with permission from Elsevier.

No increase in uterine weight or obvious changes in uterine histology occur between 4 and 8 hr (Figures 1 and 2). Nevertheless, our data reveal that this phase is associated with the induction of a large cluster of genes (Figure 5). Most are induced 2 hr after E_2_ administration, reach maximal expression at 4 or 8 hr, and return to control or subcontrol levels by 48 hr (Figure 5B). Most of these genes play roles in mRNA and protein synthesis, demonstrating that the bulk of transcriptional activity occurring at this time functions to increase the capacity of the uterus for new protein synthesis. This is consistent with earlier observations that exposure to E_2_ results in a rapid increase in the mRNA and protein content of the uterus (Clark and Mani 1994). Our data define the molecular basis for these prior observations and identify the genes targeted by ERs to induce these effects.

In a broad sense, protein synthesis includes the interlinked processes of transcription, mRNA processing, mRNA export into the cytoplasm, protein translation, and protein folding (Orphanides and Reinberg 2002, and references therein; Figure 5G). Our data reveal the coordinated induction of genes involved in each of these processes (Figure 5A–F). These genes include *a*) components of the RNAP II general transcription machinery (*RPB8*, *RPB10, TAF10*; Figure 5A); *b*) transcription termination and polyadenylation factors (*NSAP1*; Figure 5A); *c*) mRNA splicing factors (*SFPQ, U2AF1, RNPS1*; Figure 5A); *d*) mRNA export proteins (*NXF1*; Figure 5C); *e*) protein translation factors (*EIF1A, EIF2A, EIF2B, EIF3*; ribosomal proteins *RPL11, RPL12, RPL20, RPL52*, *RPS18b*, and tRNA synthetases *VALRS*, *GLURS, PHERS*; Figure 5D), and *f* ) protein folding factors (*FKBP4*, *CCT3, CCT6a, CCT7, CCT8*; Figure 5E). The down-regulation of several genes associated with transcriptional repression (*HDA1, TGIF, MAD4, EZH1*) and mRNA degradation (*AUH*; Figure 5B) may also contribute to the general elevation of mRNA synthesis. We also note a concurrent increase in the expression of components of the ubiquitin–proteasome proteolytic pathway (*PAD1, SUG1*; Figure 5F) and genes whose products are required for the nuclear import and export of proteins (*IMPORTIN*α*2, IMPORTIN*α*3*, *RAE1, G3BP2*; Figure 5C), indicating that E_2_ additionally elevates proteasome levels and nuclear-cytoplasmic protein transport activity at this time. We conclude that E_2_ is able to increase protein synthesis activity in the uterus by altering the expression of genes involved in all aspects of the protein biosynthesis pathway.

**Figure 6:**
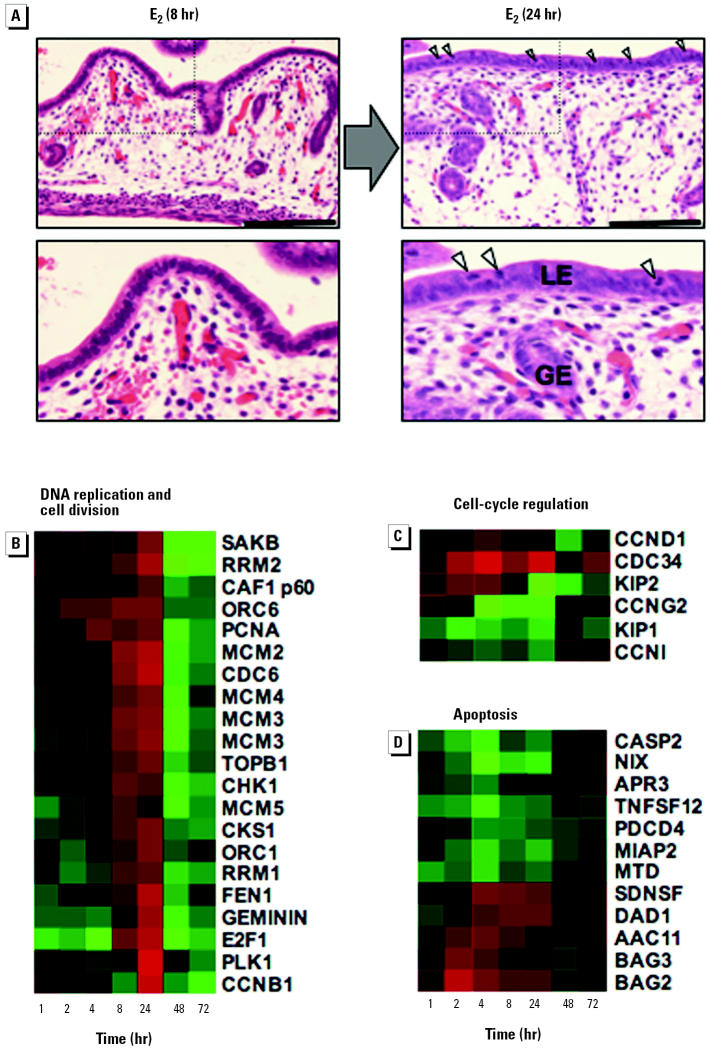
Phase 3: coordinated regulation of genes controlling chromosome replication and the cell cycle. (*A*) Thickening of luminal (LE) and glandular epithelium (GE) and increased number of mitotic cells (indicated by arrowheads) between 8 and 24 hr after a single dose of E_2_. Longitudinal 0.3-μm–thick paraffin sections of uteri were stained with hematoxylin and eosin. Scale bar, 50 μm. Coordinated expression of genes involved in (*B*) chromosome replication and cell division, (*C*) cell-cycle regulation, (*D*) and apoptosis. Gene trees were generated as described in Figure 4. Data derived from independent Affymetrix probe sets are shown for *MCM3*. See Appendix for gene nomenclature and Affymetrix probe sets.

Therefore, during the first two phases of the transcriptional program, E_2_ induces the expression of a battery of sequence-specific transcriptional regulators (phase 1; Figure 4C) and then induces the expression of genes in the protein synthesis pathway (phase 2; Figure 5). It appears, therefore, that, during phase 1, E_2_ specifies the gene expression networks that will be active, and then ensures during phase 2 that these networks have sufficient mRNA and protein synthesis capacity to operate. In addition the increased expression of components of the RNA and protein synthesis machinery is likely to be a prerequisite for proliferation in the uterus because cells must increase their mass before division to provide sufficient cellular components required for survival of the daughter cells (Norbury and Nurse 1992). Consistent with this, we note that induction of protein synthesis components immediately precedes the up-regulation of genes required for proliferation (Figure 6; see below). An additional function of the increased uterine capacity for protein synthesis may be to facilitate the production of the abundant cytoarchitectural and secreted proteins induced at the end of the uterotrophic response (see below).

### Phase 3: Coordinated Regulation of Genes Controlling Chromosome Replication and the Cell Cycle

The next phase in the uterotrophic response occurs between 8 and 24 hr and involves an approximate doubling in uterine weight (Figure 1A) and a large increase in the thickness of the luminal epithelium (Figures 2C, 6A). A quantitative histologic analysis of mitotic figures in the uterine cells (“Materials and Methods”) revealed a clear and statistically significant (*p* < 0.01) increase with E _2_ at 24 hr, whereas no E_2_-dependent increase was observed at 8, 48, or 72 hr (Table 1, Figure 6A). These observations are consistent with previous studies showing that most cells in the immature rodent uterus are stimulated to leave their quiescent state and divide synchronously under the influence of E_2_ (Kaye et al. 1971; Quarmby and Korach 1984).

We found that genes required for the replication of chromosomal DNA (*PCNA*, *FEN1, CDC6, MCM2, MCM3, MCM4, MCM5, ORC1, ORC6, RRM1, RRM2*) and genes required for postreplicative phases of the cell division cycle (e.g., *CCNB1, PLK1*) are coordinately induced and reach maximal expression levels between 8 and 24 hr (Figure 6B), consistent with the timing of the histologic changes observed in Figure 6A. Genes required for maintaining genome integrity (*CHK1, CKS1, GEMININ*) and the epigenetic status of newly replicated DNA (*CAF-1 p60, AHCY*) are also up-regulated at 8 and/or 24 hr (Figure 6B). It is striking that after their induction during the proliferative phase (8–24 hr), the expression levels of most genes that regulate chromosome replication and cell division are reduced to levels well below those of control animals (Figure 6B). This suggests that mechanisms exist for the active repression of these genes to prevent further rounds of proliferation. Declining E_2_ levels in mice 48 hr after a single subcutaneous injection may also contribute to the cessation of proliferation. Together, these data provide a molecular explanation for the changes in uterine weight and histology that occur between 8 and 24 hr (Figures 1A, 2, and 6A) and support the assertion that the early increase in weight seen at 4 hr is due to fluid uptake. Furthermore, these gene expression changes demonstrate that cell proliferation is restricted to a narrow window of time between 8 and 24 hr by the coordinated regulation of chromosome replication and cell division genes.

### Regulation of Cell Division

Our data also provide insight into the mechanisms by which E_2_ releases cells of the immature uterus from quiescence and promotes cell division. The E_2_-induced expression profile of *E2F1*, a key transcriptional regulator of DNA replication genes (Ohtani 1999), closely parallels the induction of the chromosome replication genes (Figure 6B), consistent with the proposal that *E2F1* regulates the expression of components of the DNA replication fork in human breast cancer cell lines exposed to E_2_ (Lobenhofer et al. 2002). Our data indicate that release from quiescence also involves the E_2_-induced down-regulation of genes that maintain cells in a growth-arrested state (*KIP1*, *CCNG2, CCNG1*). The principle way in which mitogens induce proliferation of quiescent cells involves a reduction in levels of the Kip1 protein, which inhibits the activities of cyclin–cdk complexes and induces cell cycle arrest (Olashaw and Pledger 2002). We found that *KIP1* was down-regulated within 1 hr of E_2_ exposure and remains repressed over a period of at least 24 hr, only reaching control levels when cell proliferation has ceased (Figure 6C). Furthermore, E_2_ may promote degradation of the Kip1 protein via the induction of *CDC34* (Figure 6C), a gene that has been implicated in the ubiquitin-mediated degradation of Kip1 (Koepp et al. 1999). These data suggest that E_2_ promotes cell proliferation by coordinately reducing Kip1 mRNA and protein levels. It is not clear whether *KIP1* is a direct or indirect target of the activated ERs. However, *KIP1* gene expression is controlled by ras-mediated PI3K signaling pathways (Olashaw and Pledger 2002), components of which are up-regulated rapidly in response to E_2_ (e.g., *DEXRAS1*, *RASSF1*; Figure 4B).

### Suppression of Apoptosis

E_2_ protects against apoptosis in a number of tissues, including brain, testes, and uterus (Thompson 1994). Although the anti-apoptotic activity of estrogen in the uterus is thought to play a crucial role in the maintenance of uterine homeostasis, the mechanistic basis for this action has not been defined. Our data reveal that E_2_ induces the expression of anti-apoptotic genes (*BAG2, BAG3, DAD1*) while simultaneously down-regulating the expression of pro-apoptotic genes (*CASP2*, *NIX*; Figure 6D). Thus, apoptosis appears to be suppressed through transcriptional mechanisms during E_2_-induced uterine growth. Consistent with these observations, E_2_ also induces the apoptotic regulators *BCL2* and *BAG1* in cultured breast cancer cells (Perillo et al. 2000; Soulez and Parker 2001). It will be important to determine whether estrogens elicit similar changes in the expression of apoptosis-regulating genes in other tissues.

### Phase 4: Induction of Genes Involved in Uterine Cell Differentiation and Defense Responses

The period from 24 to 72 hr after E _2_ exposure is associated with remodeling of the luminal epithelial cell layer, including the formation of secretory epithelial cells and a glycocalyx layer consisting of glycoproteins (Paria et al. 2003; Weitlauf 1994). These changes result in the formation of a highly differentiated epithelial layer that is primed for cell recognition and adhesion events necessary for embryo attachment and implantation.

### Changes in Cytoarchitecture

**Figure 7:**
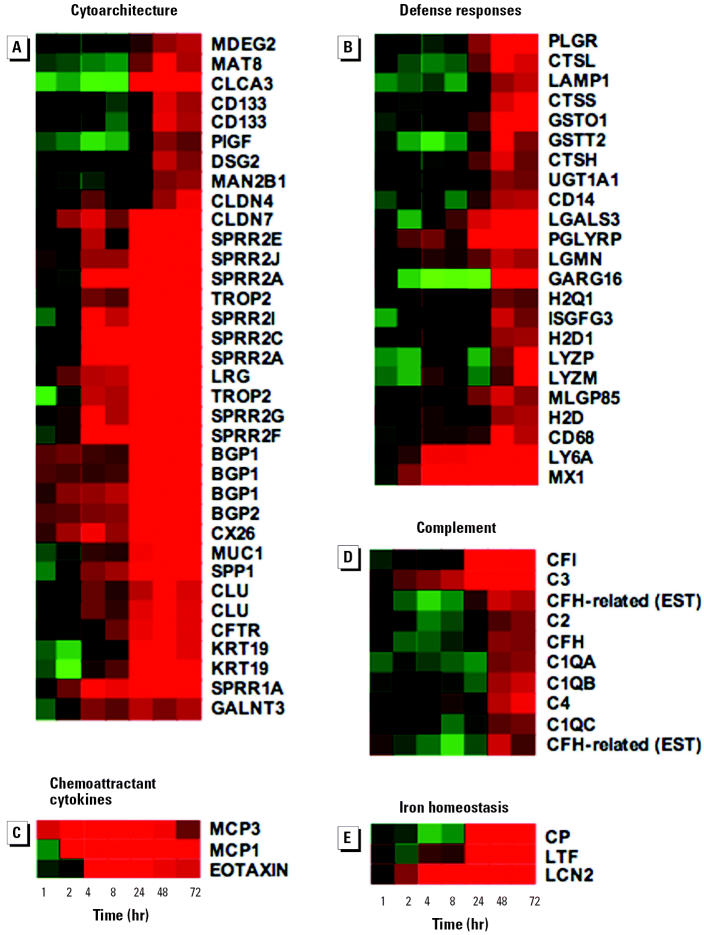
Phase 4: induction of genes involved in uterine cell differentiation and defense responses. (*A*) Cytoarchitecture; (*B*) defense responses; (*C*) chemoattractant cytokines; (*D*) complement; and (*E*) iron homeostasis. Gene trees were generated as described in Figure 4. Data derived from independent Affymetrix probe sets are shown for *SPRR2A*, *CD133*, *TROP2*, *BGP1*, *CLU*, *KRT19*, and *CFH*. Detailed quantitative data for the SPRR gene family are shown in Figure 8B. See Appendix for gene nomenclature and Affymetrix probe sets.

The final phase of the uterotrophic response coincides with the induction of a battery of genes involved in the cytoarchitectural remodeling of proliferating uterine cells, thus providing a further link between phenotypic and gene expression changes (Figure 7A). These genes encode components of desmosomes (*DSG2*), gap junctions (*CX26*), tight junctions (*CLDN4, CLDN7*), the cornified envelope (*SPRR1A, 2A, 2B, 2E, 2F, 2G, 2I, 2J*), intermediate filaments (*KRT19*), and a variety of cell-surface and extracellular-matrix glycoproteins (*SPP1, BGP1, BGP2, MUC1, TROP2, CLU*). The latter class of genes is likely to contribute to the formation of the glycocalyx layer present on differentiated uterine epithelium (Weitlauf 1994). The concomitant E_2_-dependent induction of a number of enzymes required for carbohydrate metabolism (*MAN2B1, GALNT3*) may provide the increase in sugar metabolism necessary for the production of these glycoproteins. E_2_ also induces genes encoding ion channels that regulate the balance of Na^+^ absorption and Cl^−^ secretion across the endometrial epithelium to maintain a luminal fluid microenvironment suitable for implantation (*CFTR, CLCA3, MAT8*; Figure 7A).

### Defense Responses

A number of genes involved in host defense processes or detoxification are first regulated between 24 and 72 hr (Figure 7B). We speculate that the products of these genes may provide an environment that is protective of, and facilitates, embryo implantation and development. These include genes encoding lysosomal enzymes (*LYZP, LYZM, CTSH CTSL, CTSS, LGMN*), genes involved in detoxification and clearance of xenobiotics (*GSTO1, GSTT2, UGT1A1*), and genes involved in immune and inflammatory responses (*CD14, MX1, PIGR*). The up-regulation of genes encoding chemoattractant cytokines (Figure 7C) for infiltrating eosinophils (*EOTAXIN*) and monocytes (*MCP1*/*3*) is consistent with previous observations of immune cell infiltration into the uterus (Gouon-Evans and Pollard 2001, and references therein). Another E_2_-regulated defense response may be provided by the induction of LTF (Liu and Teng 1992), an iron-binding protein with bacteriostatic activity (Singh et al. 2002). Our data reveal the induction of two additional iron metabolism genes at this time (*CP, LCN2*; Figure 7E; Kaplan 2002), suggesting a role for iron homeostasis in the uterotrophic response to E_2_.

Several components of the complement system are also induced 48–72 hr after exposure to E _2_. These include *C1QA, C1QB, C1QC, C2, C3, C4, CFH,* and *CFI* (Figure 7D). Although many complement components have been identified in female reproductive epithelium, only *C3* has previously been established as an E_2_-responsive gene (Sundstrom et al. 1989). In addition to participating in immune and inflammatory responses and host resistance, there is increasing evidence that complement functions in tissue remodeling and organ regeneration (Mastellos and Lambris 2002). Intriguingly, complement also influences mammalian reproduction and particularly the integrity of maternofetal interfaces during pregnancy (Caucheteux et al. 2003; Mastellos and Lambris 2002). Therefore, it is possible that the complement system may play a noninflammatory role in the uterotrophic response.

### Evidence for a Transcriptional Cascade in the Uterus

**Figure 8:**
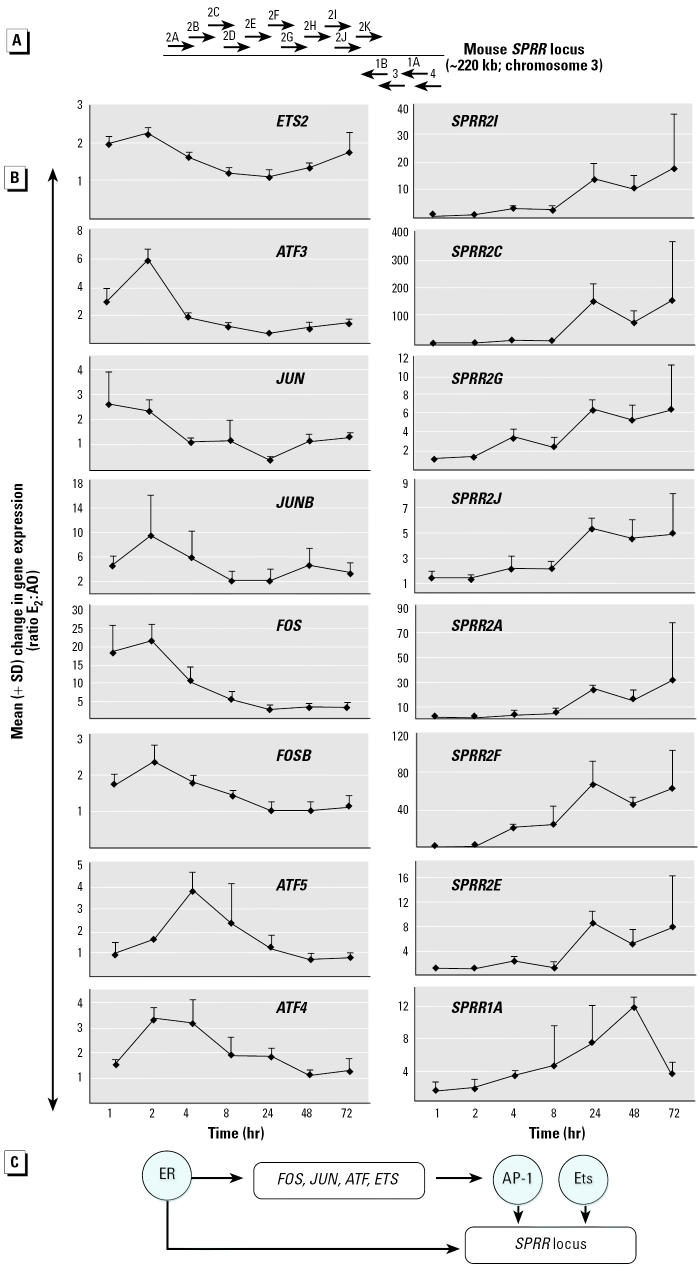
Evidence for a transcriptional regulatory network during the uterotrophic response. (*A*) Organization of mouse SPRR genomic locus that is coordinately regulated by the transcription factors AP-1 and Ets. (*B*) E_2_-induced expression (mean + SD) of genes encoding AP-1 and Ets transcription factors temporally precedes the coordinate regulation of the tandem array of SPRR genes. (*C*) Feed-forward model for an ER-dependent transcriptional cascade in the uterus. Transcriptional regulators are represented by blue circles. Gene promoters are represented by white rectangles. See Appendix for gene nomenclature and Affymetrix probe sets.

It is striking that many different induction profiles can be seen in the genes regulated by E_2_: some genes are induced within 1 hr of exposure, whereas others are not induced until 48 hr (Figure 3B). The induction of a large number of sequence-specific transcription factors during the first phase of the response suggests that a transcriptional cascade may operate in the uterus, with the products of genes induced at the beginning of the program regulating the transcription of those toward the end. The regulation of the SPRR genes provides evidence for the existence of such a cascade (Figure 8). The mouse SPRR genes are located in a tandem array at the same chromosomal locus, and their transcription is regulated by the AP-1 and Ets transcription factors (Patel et al. 2003; Figure 8A). Eight members of the SPRR gene family are induced between 4 and 72 hr, with maximal induction occurring between 24 and 48 hr (Figure 8B). Intriguingly, the mRNAs encoding Ets2 and components of AP-1 (c-Jun, JunB, c-Fos, FosB, and Atf3, Atf4, Atf5) are maximally induced during the first phase of the uterotrophic response, between 1 and 4 hr (Figure 8B). We speculate, therefore, that a transcriptional cascade operates, in which ER-αor ER-βinduces the expression of Ets2 and AP-1 components, which in turn regulate the transcription of the SPRR genes (Figure 8C). Alternatively, it is possible that ER-αor ER-βcooperates with Ets2 and AP-1 to regulate the expression of the SPRR genes. In this way, transcription of the SPRR genes would not begin until sufficient levels of Ets2 and AP-1 were present. Consistent with this model, feed-forward loops (in which a transcriptional regulator controls a second transcription factor that then functions in concert with the initial regulator on a common downstream target gene) are emerging as common mechanisms in eukaryotes for transcriptional networks (Lee et al. 2002). It is likely that analysis of the regulatory regions of other E_2_-responsive genes during the uterotrophic response will suggest the existence of additional transcriptional networks.

## Discussion

**Figure 9:**
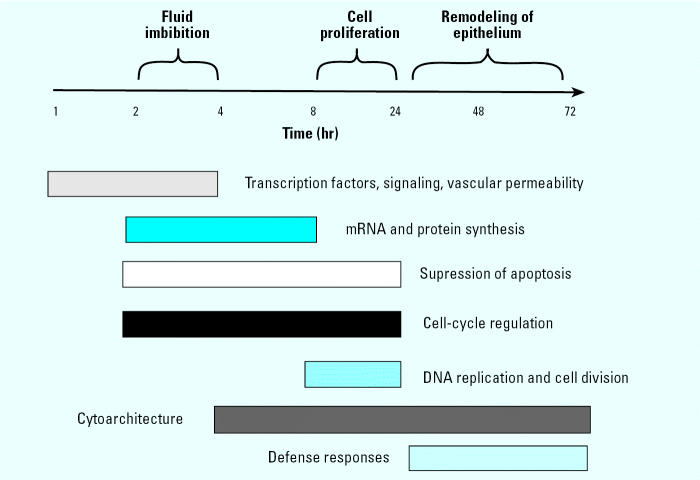
Summary of the transcriptional program associated with E_2_-induced uterine growth showing the successive regulation of genes with distinct molecular functions.

Our data describe at an unprecedented level of detail the molecular events that initiate and drive uterine physiologic changes upon exposure to the sex steroid hormone E _2_ in the immature mouse uterus. Gene expression profiling reveals that E_2_ induces a multistage and tightly coordinated transcriptional program that regulates successive and functionally interlinked cellular processes during the uterotrophic response (Figure 9). The temporal patterns of gene expression we have identified for E_2_ are consistent with, and extend, those reported recently for the uterotrophic response of immature, ovariectomized mice after exposure to 17α-ethynylestradiol (Fertuck et al. 2003), in which concordant temporal responses were seen for genes involved in several functional categories in Figure 9. These include RNA and protein metabolism, cell cycle regulation, immune responses, and complement components. Furthermore, many of the genes regulated by exogenous E_2_ in our study are also differentially regulated in response to endogenous hormones (Tan et al. 2003).

Comparison of gene expression changes with alterations in uterine weight and histologic alterations, and analysis of gene expression data according to gene function allowed us to implicate specific groups of genes in driving water imbibition in the stromal endothelium, synchronous cell proliferation, and cytoarchitectural changes associated with luminal epithelial cell differentiation. These data thus provide a detailed mechanistic view of the relationships between the uterotrophic response and the underlying transcriptional program. Furthermore, this work demonstrates that comparison of temporal changes in gene expression and conventional toxicology parameters (uterine weight and histologic changes) can provide an understanding of the relationships between gene expression patterns and phenotypic change.

E_2_ can regulate transcription through a combination of at least two distinct signaling pathways: *a*) via activation of the nuclear transcription factors ER-αand ER-β(Hall et al. 2001; McKenna and O’Malley 2002; Moggs and Orphanides 2001; Tremblay and Giguere 2002) and *b*) via extranuclear or “nongenomic” signaling events (Falkenstein et al. 2000; Hammes 2003; Moggs et al. 2003). The transcriptional responses to E_2_ that we have defined here are likely to involve a combination of direct gene regulation by nuclear ERs and indirect gene regulation via extranuclear signaling pathways. Although the uterus of the immature mouse expresses both ER subtypes (αand β) at comparable levels (Weihua et al. 2000), recent transcript profiling studies using ovariectomized ER-knockout mice revealed a predominant role for ER-αin the regulation of estrogen-responsive genes in the uterus (Hewitt et al. 2003; Watanabe et al. 2003) consistent with the observation that only a partial uterotrophic response occurs in ER-αknockout mice (Lubahn et al. 1993). Therefore, it is likely that most E_2_-responsive genes we have identified are regulated by ER-α. However, identification of the direct gene targets for each ER subtype will ultimately require the development of methods for measuring the occupancy of receptor subtypes at promoters *in vivo*. Nevertheless, our temporal analysis of E_2_-responsive genes provides novel insights into the transcriptional cascades that are initiated through E_2_-responsive transcription factors.

The molecular events described here for the reference natural estrogen E_2_ provide the basis for understanding how other estrogenic chemicals, including synthetic estrogens and phytoestrogens, induce their effects (Moggs et al. 2004). Increasing attention is being paid to the use of gene expression changes in the uterus for the identification of surrogate markers for short-term rodent estrogenicity assays (Naciff et al. 2002, 2003; Owens and Ashby 2002; Watanabe et al. 2002), and our data reveal a large number of novel candidate marker genes. The insights provided by these data, into how an ER ligand coordinates transcriptional regulatory networks that result in proliferation and differentiation in a complex organ, provide a paradigm for understanding the modes of action of other nuclear receptors.

## Supplementary Material

Supplemental Figures and Tables

## Figures and Tables

**Table 1:** Quantitative histologic analysis of mitotic figures in uterine cells after exposure to E_2_ for 8, 24, 48, and 72 hr.*^a^*

	Mitosis/mm^2^ (mean ± SD)	
Time (hr)	AO (5 mL)	E_2_ (400 μg)
8	1.36 ± 1.81	0.51 ± 0.41
24	3.86 ± 5.05	25.15 ± 6.37**
48	3.81 ± 0.83	3.46 ± 3.26
72	3.88 ± 2.28	1.67 ± 1.77

Quantitative mitotic index data were derived from four animals per group.

a Data were assessed for statistical significance using ANOVA and a two-sided Student *t*-test (see “Materials and Methods”).

** *p* < 0.01.

**Table:** **Appendix.** Gene nomenclature and Affymetrix probe sets for Figures 4–8.*^a^*

Gene symbol	Affymetrix Probe Set	Gene description
**Figure 4B —Signaling components**		
IL17R	99992_at	interleukin 17 receptor
RAP1	160822_at	Rap1, GTPase-activating protein 1
DEXRAS1	99032_at	RAS, dexamethasone-induced 1
MKP1	104598_at	dual specificity phosphatase 1
WNT4	103238_at	wingless-related MMTV integration site 4
IGFBP10	92777_at	cysteine rich protein 61
PIP92	99109_at	immediate early response 2
PIM3	96841_at	similar to serine/threonine-protein kinase pim-3
ARHU	96747_at	ras homolog gene family, member U
CISH3	162206_f_at	cytokine inducible SH2-containing protein 3
NAB2	100962_at	Ngfi-A binding protein 2
SOCS3	92232_at	cytokine inducible SH2-containing protein 3
EPLG2	98407_at	ligand for receptor tyrosine kinase ELK
IL17R	99991_at	interleukin 17 receptor
CDKN1A	98067_at	cyclin-dependent kinase inhibitor 1A (P21)
CDKN1A	94881_at	cyclin-dependent kinase inhibitor 1A (P21)
WSB1	98946_at	WD-40-repeat-containing protein with a SOCS box
VEGF	103520_at	vascular endothelial growth factor A
GADD45	102292_at	growth arrest and DNA-damage-inducible 45
SYT	99610_at	synovial sarcoma translocation, chromosome 18
SOCS1	92832_at	cytokine inducible SH2-containing protein 1
GADD45g	101979_at	growth arrest and DNA-damage-inducible 45 gamma
GLY96	94384_at	immediate early response 3
MAPKAP2	160353_i_at	MAP kinase-activated protein kinase 2
KLK22	101289_f_at	epidermal growth factor binding protein type 1
TROB	99532_at	tob family
RGS3	160747_at	regulator of G-protein signaling 3
GNA13	100514_at	guanine nucleotide binding protein, alpha 13
RAB11A	96238_at	RAB11a, member RAS oncogene family
PLGF	92909_at	placental growth factor
BDKRB1	101748_at	bradykinin B1 subtype receptor
CF3	97689_at	coagulation factor III
PDK4	102049_at	pyruvate dehydrogenase kinase, isoenzyme 4
HERPUD1	95057_at	homocysteine-inducible, endoplasmic reticulum stress-inducible, ubiquitin-like domain member 1
MYD116	160463_at	myeloid differentiation primary response gene 116
NORE1	102028_at	Ras association (RalGDS/AF-6) domain family 5
NET1A	94223_at	neuroepithelial cell transforming gene 1
GEM	92534_at	GTP binding protein (gene overexpressed in skeletal muscle)
SNRK	97429_at	SNF related kinase
ALASH	93500_at	aminolevulinic acid synthase 1
NTTP1	161171_at	dual specificity phosphatase 8
MAPKAP2	95721_at	MAP kinase-activated protein kinase 2
MEK1	92585_at	mitogen activated protein kinase kinase 1
RGSr	94378_at	regulator of G-protein signaling 16
RASSF1	102379_at	Ras association (RalGDS/AF-6) domain family 1
NGEF	93178_at	neuronal guanine nucleotide exchange factor
C-KIT	99956_at	kit oncogene
NOTCH1	97497_at	Notch gene homolog 1
BTG3	96146_at	B-cell translocation gene 3
PC4	160092_at	interferon-related developmental regulator 1
SGK	97890_at	serum/glucocorticoid regulated kinase
ADM	102798_at	adrenomedullin
ANGPT2	92210_at	angiopoietin 2
UBQLN1	95601_at	ubiquilin 1
THBS1	160469_at	thrombospondin
ROCK2	98504_at	rho-associated coiled-coil forming kinase 2
SNK	92310_at	serum-inducible kinase
MAP2K3	93315_at	mitogen activated protein kinase kinase 3
ENG	100134_at	endoglin
PTDSR	95486_at	phosphatidylserine receptor
SWIP2	160296_at	WD-40-repeat-containing protein with a SOCS box
AKT	100970_at	thymoma viral proto-oncogene 1
RHOC	96056_at	ras homolog gene family, member C
TGFB2	93300_at	transforming growth factor, beta 2
EPCR	98018_at	protein C receptor, endothelial
KLK6	100061_f_at	kallikrein 6
GALN	100407_at	galanin
NEDD4B	103907_at	neural precursor cell expressed, developmentally down-regulated gene 4-like
KLK22	95775_f_at	kallikrein 22
KLK9	94716_f_at	kallikrein 9
MCP1	102736_at	platelet-derived growth factor-inducible protein JE
TIE1	99936_at	tyrosine kinase receptor 1
RAMP1	104680_at	receptor (calcitonin) activity modifying protein 1
PGF	97769_at	prostaglandin F receptor
PDGFαRA	95079_at	platelet derived growth factor receptor, alpha polypeptide
OB-RGRP	93600_at	leptin receptor
ERK1	101834_at	mitogen activated protein kinase 3
GRB7	103095_at	growth factor receptor bound protein 7
ADCY6	102321_at	adenylate cyclase 6
TIE1	161184_f_at	tyrosine kinase receptor 1
GNAI1	104412_at	guanine nucleotide binding protein, alpha inhibiting 1
ADCY7	103392_at	adenylate cyclase 7
TIE2	102720_at	endothelial-specific receptor tyrosine kinase
GPCR26	100435_at	endothelial differentiation, lysophosphatidic acid G-protein-coupled receptor, 2
**Figure 4C—Transcription factors**		
GIF	99603_g_at	TGFB inducible early growth response
GIF	99602_at	TGFB inducible early growth response
ETS2	94246_at	E26 avian leukemia oncogene 2, 3’ domain
ID1	100050_at	inhibitor of DNA binding 1
SMAD7	92216_at	MAD homolog 7
C-JUN	100130_at	Jun oncogene
BRF2	160273_at	zinc finger protein 36, C3H type-like 2
IRF8	98002_at	interferon concensus sequence binding protein
AGP/EBP	92925_at	CCAAT/enhancer binding protein (C/EBP), beta
C-FOS	160901_at	c-fos oncogene
KROX24	98579_at	zinc finger protein Krox-24
FOSB	103990_at	FBJ osteosarcoma oncogene B
NR4A1	102371_at	N10 nuclear hormonal binding receptor
SOX18	161025_f_at	SRY-box containing gene 18
SOX18	104408_s_at	SRY-box containing gene 18
KROX20	102661_at	Early growth response 2
ESG	104623_at	transducin-like enhancer of split 3, homolog of Drosophila E(spl)
FOG	97974_at	zinc finger protein, multitype 1
NCOR2	95129_at	nuclear receptor co-repressor 2
SOX11	101631_at	SRY-box containing gene 11
C/EBP	94466_f_at	CCAAT/enhancer binding protein alpha (C/EBP), related sequence 1
PRX2	103327_at	paired related homeobox 2
ATF4	100599_at	activating transcription factor 4
STAT5B	100422_i_at	signal transducer and activation of transcription 5A
HEY1	95671_at	hairy/enhancer-of-split related with YRPW motif 1
ATF5	103006_at	activating transcription factor 5
C/EBP	98447_at	CCAAT/enhancer binding protein
RIP140	103288_at	nuclear receptor interacting protein 1
CRTR1	103761_at	Tcfcp2-related transcriptional repressor 1
MEF2A	93852_at	myocyte enhancer factor 2A
TIS11	92830_s_at	zinc finger protein 36
STAT5B	100423_f_at	signal transducer and activation of transcription 5A
ATF3	104155_f_at	activating transcription factor 3
CART1	100005_at	TNF receptor associated factor 4
JUNB	102362_i_at	transcription factor junB
**Figure 5A—RNA synthesis**		
SFPQ	99621_s_at	splicing factor proline/glutamine rich (polypyrimidine tract binding protein associated)
U2AF1	97486_at	U2 small nuclear ribonucleoprotein auxiliary factor (U2AF), 35 kDa
RBMXP1	160192_at	RNA binding motif protein, X chromosome retrogene
DDX21	94361_at	DEAD/H (Asp-Glu-Ala-Asp/His) box polypeptide 21 (RNA helicase II/Gu)
DDX3	101542_f_at	DEAD (aspartate-glutamate-alanine-aspartate) box polypeptide 3
NSAP1	94985_at	NS1-associated protein 1
MKI67 bp	93342_at	Mki67 (FHA domain) interacting nucleolar phosphoprotein
ELAVL1	94001_at	ELAV (embryonic lethal, abnormal vision, Drosophila)-like 1 (Hu antigen R)
PSP1	103393_at	paraspeckle protein 1
SRP20	101003_at	splicing factor, arginine/serine-rich 3 (SRp20)
JKTBP	96084_at	heterogeneous nuclear ribonucleoprotein D-like
RPA2	92225_f_at	RNA polymerase 1–2 (128 kDa subunit)
RALY	98511_at	hnRNP-associated with lethal yellow
SFRS10	95791_s_at	splicing factor, arginine/serine-rich 10
FBL	160503_at	fibrillarin
SNRPA1	101506_at	small nuclear ribonucleoprotein polypeptide A’
TASR	98048_at	neural-salient serine/arginine-rich
RPB10	93551_at	RNA polymerase II subunit 10
AUF1	94303_at	heterogeneous nuclear ribonucleoprotein D
HRMT1L2	96696_at	heterogeneous nuclear ribonucleoproteins methyltransferase-like 2
CGI-110	95714_at	pre-mRNA branch site protein p14
SMN	103620_s_at	survival motor neuron
RPB8	97254_at	RNA binding motif protein
RNPS1	93518_at	ribonucleic acid binding protein S1
NCL	160521_at	nucleolin
RPA1	93620_at	RNA polymerase 1–4 (194 kDa subunit)
HNRPA2B1	93118_at	heterogeneous nuclear ribonucleoprotein A2/B1
SNRPD1	100577_at	small nuclear ribonucleoprotein D1
H/ALAsnRNP	97824_at	nucleolar protein family A, member 2
TAF10	103910_at	TAFII30
DDX24	99096_at	DEAD/H (Asp-Glu-Ala-Asp/His) box polypeptide 13 (RNA helicase A)
**Figure 5B**		
MAD4	99024_at	Max dimerization protein 4
EZH1	100486_at	enhancer of zeste homolog 1 (Drosophila)
HDA1	104376_at	histone deacetylase 5
AUH	96650_at	AU RNA binding protein/enoyl-coenzyme A hydratase
TGIF	101502_at	TG interacting factor
**Figure 5C—Nuclear import/export**		
POM121	96174_at	nuclear pore membrane protein 121
NXF1	101079_at	nuclear RNA export factor 1 homolog (*S. cerevisiae*)
IMPORTINa3	96010_at	karyopherin (importin) alpha 3
RAE1	160466_at	RNA export 1 homolog (S. pombe)
IMPORTINa2	92790_at	karyopherin (importin) alpha 2
G3BP2	94913_at	Ras-GTPase-activating protein (GAP120) SH3-domain binding protein 2
**Figure 5D—Protein translation**		
eIF3S7	99101_at	eukaryotic translation initiation factor 3, subunit 7 (zeta, 66/67kDa)
eIF2B	160365_at	eukaryotic translation initiation factor 2, subunit 2 (beta, 38kDa)
eIF3S4	96883_at	eukaryotic translation initiation factor 3, subunit 4 (delta, 44kDa)
EBNA1-bp2	96297_at	EBNA1 binding protein 2
GLNRS	96628_at	glutamyl-prolyl-tRNA synthetase
NAT1	100535_at	eukaryotic translation initiation factor 4, gamma 2
eIF3S9	93973_at	eukaryotic translation initiation factor 3, subunit 9
RPS18b	95159_at	ribosomal protein S18b
VALRS	97894_at	valyl-tRNA synthetase 2
RPL12	160431_at	mitochondrial ribosomal protein L12
eIF1A	93058_at	eukaryotic translation initiation factor 1A
eRF1	160451_at	translation releasing factor eRF1
eIF1A	103708_at	eukaryotic translation initiation factor 1A
eIF6	94826_at	integrin beta 4 binding protein
eRF1	98608_at	translation releasing factor eRF1
RPL20	94875_at	mitochondrial ribosomal protein L20
PHERS	94494_at	phenylalanine-tRNA synthetase-like
ASNS	95133_at	asparagine synthetase
eIF3S10	94250_at	eukaryotic translation initiation factor 3
NOP56	95109_at	nucleolar protein 5A
eIF2AS1	94253_at	eukaryotic translation initiation factor 2A
RRS1	96778_at	regulator for ribosome resistance homolog (S. cerevisiae)
eRF1	96755_at	translation releasing factor eRF1
eRF1	96754_s_at	translation releasing factor eRF1
SUI1	92855_at	suppressor of initiator codon mutations, related sequence 1 (*S. cerevisiae*)
RPL11	98876_at	mitochondrial ribosomal protein L11
RPL52	97443_at	mitochondrial ribosomal protein L52
**Figure 5E—Protein folding**		
CCT3	98153_at	chaperonin subunit 3 (gamma)
FKBP4	92808_f_at	FK506 binding protein 4 (59 kDa)
CCT7	160562_at	chaperonin subunit 7 (eta)
PPID	97445_at	peptidylprolyl isomerase D (cyclophilin D)
CCT10	92829_at	heat shock 10 kDa protein 1 (chaperonin 10)
CCT8	160102_at	chaperonin subunit 8 (theta)
CCT6A	162279_f_at	chaperonin subunit 6a (zeta)
CCT3	161238_f_at	chaperonin subunit 3 (gamma)
**Figure 5F—Protein degradation**		
PAD1	97274_at	26S proteasome-associated pad1 homolog
PSMB5	101558_s_at	proteasome (prosome, macropain) subunit, beta type 5
PSMD4	94302_at	proteasome (prosome, macropain) 26S subunit, non-ATPase, 4
PSMB3	94025_at	proteasome (prosome, macropain) subunit, beta type 3
SUG1	160534_at	protease (prosome, macropain) 26S subunit, ATPase 5
PSMB6	101992_at	proteasome (prosome, macropain) subunit, beta type 6
PSMB2	94219_at	proteasome (prosome, macropain) subunit, beta type 2
**Figure 6B—DNA replication and cell division**		
SAKB	98996_at	serine/threonine kinase 18
RRM2	102001_at	ribonucleotide reductase M2
CAF1 p60	100890_at	chromatin assembly factor, p60 subunit
ORC6	95712_at	origin recognition complex, subunit 6-like (S. cerevisiae)
PCNA	101065_at	proliferating cell nuclear antigen
MCM2	93112_at	mini chromosome maintenance deficient 2
CDC6	103821_at	cell division cycle 6 homolog (S. cerevisiae)
MCM4	93041_at	mini chromosome maintenance deficient 4 homolog
MCM3	160496_s_at	mini chromosome maintenance deficient (S. cerevisiae)
MCM3	100062_at	mini chromosome maintenance deficient (S. cerevisiae)
TOPB1	103071_at	topoisomerase (DNA) II binding protein
CHK1	103064_at	checkpoint kinase 1 homolog (S. pombe)
MCM5	100156_at	mini chromosome maintenance deficient 5
CKS1	97468_at	CDC28 protein kinase 1
ORC1	92458_at	origin recognition complex, subunit 1-like (S. cerevisiae)
RRM1	100612_at	ribonucleotide reductase M1
FEN1	97327_at	flap structure specific endonuclease 1
GEMININ	160069_at	geminin
E2F1	102963_at	E2F transcription factor 1
PLK1	93099_f_at	polo-like kinase homolog (Drosophila)
CCNB1	160159_at	cyclin B1, related sequence 1
**Figure 6C—Cell-cycle regulators**		
CCND1	94232_at	cyclin D1
CDC34	94048_at	cell division cycle 34 homolog
KIP2	95471_at	cyclin-dependent kinase inhibitor 1C (P57)
CCNG2	98478_at	cyclin G2
KIP1	161010_r_at	cyclin-dependent kinase inhibitor (p27)
CCNI	94819_f_at	cyclin I
**Figure 6D—Apoptosis**		
CASP2	99049_at	caspase 2
NIX	96255_at	BCL2/adenovirus E1B 19 kDa-interacting protein 3-like
APR3	160271_at	apoptosis related protein APR3
TNFSF12	93917_at	tumor necrosis factor (ligand) superfamily, member 12
PDCD4	103029_at	programmed cell death 4
MIAP2	102734_at	baculoviral IAP repeat-containing 3
MTD	98031_at	Bcl-2-related ovarian killer protein
SDNSF	97451_at	neural stem cell derived neuronal survival protein
DAD1	96008_at	defender against Apoptotic Death 1
AAC11	101035_at	apoptosis inhibitor 5
BAG3	96167_at	Bcl2-associated athanogene 3
BAG2	161129_r_at	similar to BAG-family molecular chaperone regulator-2
**Figure 7A—Cytoarchitecture**		
MDEG2	99910_at	amiloride-sensitive cation channel 1, neuronal (degenerin)
MAT8	103059_at	FXYD domain-containing ion transport regulator 3
CLCA3	162287_r_at	chloride channel calcium activated 3
CD133	93389_at	prominin
CD133	93390_g_at	prominin
PIGF	104725_at	ras-like protein
DSG2	104480_at	desmoglein 2
MAN2B1	99562_at	mannosidase 2, alpha B1
CLDN4	101410_at	claudin 4
CLDN7	99561_f_at	claudin 7
SPRR2E	100723_f_at	small proline-rich protein 2E
SPRR2J	101755_f_at	small proline-rich protein 2J
SPRR2A	101025_f_at	small proline-rich protein 2A
TROP2	103648_at	tumor-associated calcium signal transducer 2
SPRR2I	95794_f_at	small proline-rich protein 2I
SPRR2C	101761_f_at	small proline-rich protein 2C
SPRR2A	101024_i_at	small proline-rich protein 2A
LRG	97420_at	leucine-rich alpha-2-glycoprotein
TROP2	160651_at	tumor-associated calcium signal transducer 2
SPRR2G	101754_f_at	small proline-rich protein 2G
SPRR2F	94120_s_at	small proline-rich protein 2F
BGP1	102805_at	CEA-related cell adhesion molecule 1
BGP1	102804_at	CEA-related cell adhesion molecule 1
BGP1	102806_g_at	CEA-related cell adhesion molecule 1
BGP2	101908_s_at	CEA-related cell adhesion molecule 2
CX26	98423_at	connexin 26
MUC1	102918_at	mucin 1, transmembrane
SPP1	97519_at	secreted phosphoprotein 1
CLU	161294_f_at	clusterin
CLU	95286_at	clusterin
CFTR	94757_at	cystic fibrosis transmembrane conductance regulator homolog
KRT19	92550_at	keratin complex 1, acidic, gene 19
KRT19	102121_f_at	keratin complex 1, acidic, gene 19
SPRR1A	160909_at	small proline-rich protein 1A
GALNT3	162313_f_at	UDP-N-acetyl-alpha-D-galactosamine:polypeptide N-acetylgalactosaminyltransferase 3
**Figure 7B—Defense responses**		
PLGR	99926_at	polyimmunoglobulin receptor
CTSL	101963_at	cathepsin L
LAMP1	100136_at	lysosomal membrane glycoprotein 2
CTSS	98543_at	cathepsin S
GSTO1	97819_at	glutathione S-transferase omega 1
GSTT2	104603_at	glutathione S-transferase, theta 2
CTSH	94834_at	cathepsin H
UGT1A1	99580_s_at	UDP glycosyltransferase 1 family, polypeptide A6
CD14	98088_at	CD14 antigen
LGALS3	95706_at	lectin, galactose binding, soluble 3
PGLYRP	104099_at	peptidoglycan recognition protein
LGMN	93261_at	legumain
GARG16	100981_at	interferon-induced protein with tetratricopeptide repeats
H2Q1	99378_f_at	MHC beta-2-microglobulin
ISGFG3	103634_at	interferon dependent positive acting transcription factor 3 gamma
H2D1	101886_f_at	histocompatibility 2, D region locus 1
LYZP	101753_s_at	P lysozyme structural
LYZM	100611_at	lysozyme M
MLGP85	101389_at	scavenger receptor class B, member 2
H2D1	97540_f_at	histocompatibility 2, D region locus 1
CD68	103016_s_at	CD68 antigen
LY6A	93078_at	lymphocyte antigen 6 complex, locus A
MX1	98417_at	myxovirus (influenza virus) resistance 1
**Figure 7C—Chemoattractant cytokines**		
MCP3	94761_at	monocyte chemoattractant protein 3
MCP1	102736_at	platelet-derived growth factor-inducible protein JE
EOTAXIN	92742_at	small inducible cytokine a11
**Figure 7D—Complement**		
CFI	99927_at	complement component factor i
C3	93497_at	complement component 3
CFH-related	92291_f_at	complement component factor-related
C2	103673_at	complement component 2 (within H-2S)
CFH-related	101853_f_at	complement component factor h
C1QA	98562_at	complement component 1, q subcomponent, alpha polypeptide
C1QB	96020_at	complement component 1, q subcomponent, beta polypeptide
C4	103033_at	complement component 4 (within H-2S)
C1QC	92223_at	complement component 1, q subcomponent, c polypeptide
CFH-related	94743_f_at	complement component factor-related
**Figure 7E—Iron homeostasis**		
CP	92851_at	ceruloplasmin
LTF	101115_at	lactotransferrin
LCN2	160564_at	lipocalin 2/24p3 gene.
**Figure 8B**		
ETS2	94246_at	E26 avian leukemia oncogene 2, 3’ domain
ATF3	104155_f_at	activating transcription factor 3
JUN	100130_at	Jun oncogene
JUNB	102362_i_at	transcription factor junB
FOS	160901_at	c-fos oncogene
FOSB	103990_at	FBJ osteosarcoma oncogene B
ATF5	103006_at	activating transcription factor 5
ATF4	100599_at	activating transcription factor 4
SPRR2I	95794_f_at	small proline-rich protein 2I
SPRR2C	101761_f_at	small proline-rich protein 2C
SPRR2G	101754_f_at	small proline-rich protein 2G
SPRR2J	101755_f_at	small proline-rich protein 2J
SPRR2A	101025_f_at	small proline-rich protein 2A
SPRR2F	94120_s_at	small proline-rich protein 2F
SPRR2E	100723_f_at	small proline-rich protein 2E
SPRR1A	160909_at	small proline-rich protein 1A

a Gene annotations were derived by interrogation of the NetAffx (Liu et al. 2003) database; http://www.affymetrix.com/analysis/index.affx and by homology searching of nucleotide sequence databases (BLASTn; http://www.ncbi.nih.gov/BLAST/) using Affymetrix probe target sequences.
