# Supplementary material for: Phenotypic Anchoring of Gene Expression Changes during Estrogen-Induced Uterine Growth
Source: Environ Health Perspect. 2004 Oct 7;112(16):1589–606. doi: 10.1289/txg.7345 (PMC1247656; doi:10.1289/txg.7345)
Supplement: Supplemental Figures and Tables [file ehp0112-001589s1.pdf]

# Supplementary Figure 1

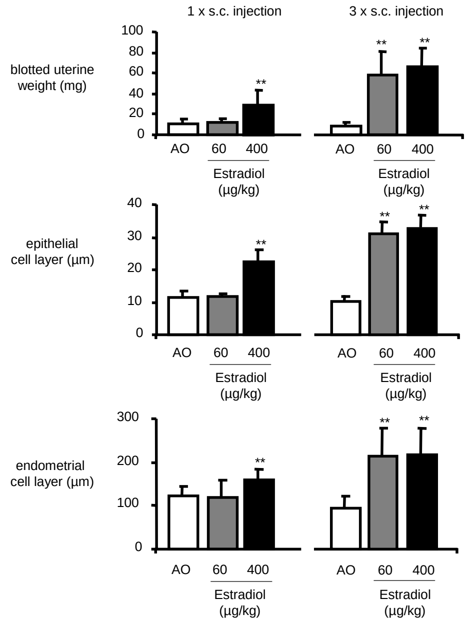


Supplementary Figure 1.
Comparison of single and multiple doses of $E_2$ (60 µg/kg versus 400 µg/kg) in the uterotrophic assay. A single s.c. dose of $E_2$ (400 µg/kg) was required to induce an increase in uterine wet weight after 72 hr compared to the lower dose (e.g. 60 µg/kg) required in standard uterotrophic assay exposure regime (i.e. repeated administration on 3 consecutive days). Data shown are mean + SD from 10 immature female mice in each treatment group. $P$<0.01 (**).

Supplementary Table 2. Major Gene Ontology associations of 3,538 E2-responsive genes*.

| GO IDs | GO Annotation | Significant children in branch | Count | Total | P-Value |
|---|---|---|---|---|---|
| GO:0043170 | Macromolecule metabolism | | 486 | 2428 | 3.04e-20 |
| GO:0019538 | | -Protein metabolism | 410 | 2047 | 4.87e-17 |
| GO:0043039 | | -tRNA aminoacylation | 18 | 39 | 7.9e-07 |
| GO:0006418 | | -tRNA aminoacylation for protein translation | 18 | 39 | 7.9e-07 |
| GO:0043038 | | -Amino acid activation | 18 | 39 | 7.9e-07 |
| GO:0006400 | | -tRNA modification | 18 | 41 | 3.04e-06 |
| GO:0009059 | | -macromolecule biosynthesis | 126 | 602 | 6.61e-06 |
| GO:0043037 | | -translation | 42 | 149 | 1.62e-05 |
| GO:0009057 | | -macromolecule catabolism | 116 | 564 | 5.34e-05 |
| GO:0006412 | | -protein biosynthesis | 94 | 436 | 5.34e-05 |
| GO:0016126 | | -sterol biosynthesis | 11 | 17 | 6e-05 |
| GO:0007049 | Cell Cycle | | 143 | 514 | 1.31e-18 |
| GO:0008283 | | -cell proliferation | 168 | 658 | 4.87e-17 |
| GO:0000067 | | -DNA replication and chromosome cycle | 40 | 102 | 2.21e-11 |

| | | | | | |
|---|---|---|---|---|---|
| GO:0006260 | | -DNA replication | 33 | 82 | 9.28e-10 |
| GO:0000074 | | -regulation of cell cycle | 70 | 262 | 8.51e-08 |
| GO:0019752 | Carboxylic acid metabolism | | 83 | 278 | 7.28e-13 |
| GO:0006082 | | -organic acid metabolism | 83 | 278 | 7.28e-13 |
| GO:0006520 | | -amino acid metabolism | 43 | 127 | 8.58e-09 |
| GO:0006631 | | -fatty acid metabolism | 31 | 103 | 7.35e-05 |
| GO:0009308 | Amine metabolism | | 57 | 191 | 1.26e-08 |
| GO:0016125 | Sterol metabolism | | 20 | 42 | 5.95e-08 |
| GO:0008203 | | -cholesterol metabolism | 18 | 39 | 7.9e-07 |
| GO:0019219 | Regulation of nucleic acid metabolism | | 262 | 1400 | 6.73e-07 |
| GO:0045449 | | -regulation of transcription | 261 | 1395 | 6.73e-07 |
| GO:0006355 | | -regulation of transcription, DNA-dependent | 253 | 1366 | 2.44e-06 |
| GO:0009451 | RNA modification | | 21 | 49 | 6.73e-07 |
| GO:0016070 | | -RNA metabolism | 61 | 242 | 9.23e-06 |
| GO:0006350 | Transcription | | 269 | 1455 | 1.08e-06 |
| GO:0006351 | | -transcription, DNA-dependent | 258 | 1396 | 2.26e-06 |
| GO:0016049 | Cell Growth | | 21 | 53 | 4.89e-06 |
| GO:0008361 | | -regulation of cell size | 21 | 56 | 2.06e-05 |

| | | | | |
|---|---|---|---|---|
| GO:0001568 | Blood vessel development | 25 | 72 | 1.77e-05 |
| GO:0006259 | DNA metabolism | 80 | 358 | 6.15e-05 |

*GO analysis was performed using GOStat (http://gostat.wehi.edu.au; Beissbarth and Speed, Bioinformatics (2004) 20(9):1464-1465). Shown are GO terms that are significantly over-represented ($p<0.0001$, using the Benjamini and Hochberg false discovery rate multiple testing correction) in the 3538 probesets altered by oestrogen when compared to the MGI database. GO terms were clustered so that terms with the same set of genes were grouped and then ranked by p-value.

Moggs et al. Phenotypic anchoring of gene expression changes during estrogen-induced uterine growth

| Statistical analysis of microarray da | A | A | A | A | A | A | A | A | A | A | A | A | A | A |
|---|---|---|---|---|---|---|---|---|---|---|---|---|---|---|
| Affymetrix probe set ID | AO 1 hr | AO 2 hr | AO 4 hr | AO 8 hr | AO 24 hr | AO 48 hr | AO 72 hr | E2 1 hr | E2 2 hr | E2 4 hr | E2 8 hr | E2 24 hr | E2 48 hr | E2 72 hr |
| AFFX-MurIL2_at | 9.5 | 10.7 | 12.5 | 8.6 | 9.7 | 15.7 | 13.8 | 8 | 10 | 12.9 | 15.8 | 15.2 | 11.2 | 11.8 |
| AFFX-MurIL10_at | 27.2 | 77.5 | 12.6 | 26.2 | 14.4 | 68.7 | 41.1 | 32.8 | 25.3 | 66.7 | 10.7 | 54.8 | 26.3 | 39.1 |
| AFFX-MurIL4_at | 20.7 | 16.2 | 21.5 | 21.4 | 22.8 | 20.2 | 24.3 | 19.2 | 24.5 | 21.2 | 50.3 | 37.9 | 29.2 | 23.8 |
| AFFX-MurFAS_at | 95.9 | 68.5 | 66 | 66.6 | 66 | 71.8 | 34.7 | 58 | 64.1 | 50.4 | 12.2 | 52.2 | 79.4 | 73.2 |
| AFFX-BioB-5_at | 401.9 | 449.1 | 308.9 | 307.1 | 252.3 | 350.4 | 344 | 344.3 | 339.7 | 299.7 | 267.9 | 358.5 | 346.5 | 346.1 |
| AFFX-BioB-M_at | 673.6 | 589.7 | 467.4 | 440.9 | 556 | 456.8 | 541.7 | 672.2 | 640.5 | 512.8 | 553.1 | 482.9 | 483.3 | 581.7 |
| AFFX-BioB-3_at | 286.7 | 321.4 | 276 | 220.2 | 278.8 | 301.1 | 208.4 | 285.1 | 286.5 | 225.3 | 286.9 | 259.6 | 268.7 | 235.5 |
| AFFX-BioC-5_at | 927.6 | 915.6 | 746.5 | 742.7 | 875.9 | 772 | 744.1 | 850.5 | 910.8 | 714.6 | 730.9 | 734.4 | 766.6 | 810.7 |
| AFFX-BioC-3_at | 607.9 | 586.6 | 484.9 | 601.1 | 649.6 | 501.6 | 484 | 576.4 | 589.5 | 453 | 466.3 | 451.1 | 488.2 | 547.6 |
| AFFX-BioDn-5_at | 956.5 | 952.2 | 740.8 | 869.5 | 976.7 | 859.8 | 691.2 | 898.2 | 911.2 | 844.3 | 752.2 | 743.8 | 793.3 | 966.3 |
| AFFX-BioDn-3_at | 4715.3 | 5024.2 | 4328.9 | 4182 | 4474.7 | 4401.9 | 4708.3 | 5209.7 | 4448.1 | 3650.6 | 4228.1 | 4185.6 | 4268.8 | 4840.2 |
| AFFX-CreX-5_at | 8770.1 | 8816.3 | 7590.1 | 8384.9 | 8235.8 | 7322 | 6804.6 | 9128.1 | 9000.4 | 7527.9 | 6836.3 | 6343 | 7086.4 | 8477.1 |
| AFFX-CreX-3_at | 12533.1 | 14420.4 | 12179.2 | 11669.7 | 12346.3 | 11914.1 | 11536.1 | 12901.4 | 13257.6 | 10820.3 | 11951.6 | 10663.1 | 11677.5 | 12496.3 |
| AFFX-BioB-5_st | 46.1 | 46.1 | 59.1 | 43.7 | 17.9 | 18.3 | 48.8 | 36 | 24.7 | 46.5 | 41.5 | 51 | 50 | 32 |
| AFFX-BioB-M_st | 61.9 | 14.2 | 63.8 | 17.2 | 65.5 | 58.3 | 93.9 | 54.8 | 21.1 | 61.2 | 27.3 | 40.9 | 70.2 | 51.1 |
| AFFX-BioB-3_st | 20.4 | 19 | 27.4 | 19.7 | 18.8 | 16.7 | 28.4 | 25.2 | 11.8 | 19.4 | 24 | 24.2 | 21.9 | 32.9 |
| AFFX-BioC-5_st | 12.1 | 15.4 | 11.8 | 10.4 | 11.2 | 17.1 | 23.9 | 15.2 | 7 | 11.7 | 12 | 13.3 | 10.4 | 12.8 |
| AFFX-BioC-3_st | 5.3 | 3.1 | 5.2 | 5.8 | 5 | 7 | 7.6 | 6.3 | 3.9 | 6.9 | 4.7 | 10.7 | 5.8 | 5.8 |
| AFFX-BioDn-5_st | 178.3 | 83.6 | 103.8 | 117.2 | 139 | 147.5 | 193.1 | 131.1 | 25.1 | 81.3 | 119.5 | 154.5 | 81.4 | 90.3 |
| AFFX-BioDn-3_st | 138.8 | 134.8 | 92.7 | 144.4 | 161.7 | 137 | 144.6 | 195.5 | 154.8 | 92.5 | 154.2 | 152.1 | 133.8 | 170.2 |
| AFFX-CreX-5_st | 149.6 | 166.3 | 126 | 140.5 | 128.8 | 148.5 | 209.8 | 226.6 | 147 | 128.7 | 175.8 | 201.2 | 175.8 | 167 |
| AFFX-CreX-3_st | 320.3 | 295.2 | 244.1 | 321.6 | 303.5 | 281.3 | 324.8 | 319.7 | 233.7 | 262.8 | 235.7 | 318.7 | 251.8 | 331.9 |
| AFFX-DapX-5_at | 20.5 | 52.3 | 32.9 | 49.2 | 32.5 | 64.4 | 46.7 | 49 | 20.2 | 28.2 | 45.4 | 50.2 | 44.3 | 24.1 |
| AFFX-DapX-M_at | 19.6 | 27.8 | 30.8 | 32.4 | 21.5 | 49.9 | 34.6 | 40.1 | 37.5 | 24.4 | 46.3 | 32.4 | 31.1 | 22.7 |
| AFFX-DapX-3_at | 4.6 | 6.4 | 10 | 6.4 | 6.2 | 7.1 | 10.3 | 17.1 | 6.7 | 8.3 | 27.2 | 28.4 | 7.1 | 4.7 |
| AFFX-LysX-5_at | 2.2 | 3.6 | 2.9 | 3.4 | 4.2 | 3.4 | 4.4 | 1.7 | 15 | 3.3 | 4.4 | 26.5 | 11.5 | 3.4 |
| AFFX-LysX-M_at | 70.2 | 65.3 | 22.3 | 17.3 | 50.7 | 11.4 | 48.6 | 55.7 | 27.9 | 12.9 | 28.4 | 42 | 9.5 | 33.7 |
| AFFX-LysX-3_at | 12.3 | 31.4 | 38.6 | 49.7 | 29.3 | 24.2 | 26.4 | 32.5 | 18 | 22.9 | 29.8 | 39.5 | 32.9 | 40.6 |
| AFFX-PheX-5_at | 5.2 | 6.3 | 5.9 | 3.5 | 3.6 | 4.5 | 4.8 | 5 | 4.7 | 4.1 | 8.6 | 4.5 | 4.7 | 6 |
| AFFX-PheX-M_at | 4.6 | 20.7 | 4.5 | 9.3 | 8.3 | 10 | 8.5 | 2.8 | 1.7 | 3.3 | 5.9 | 2.7 | 2.2 | 8.4 |
| AFFX-PheX-3_at | 25.1 | 46.7 | 47.4 | 27.8 | 23.3 | 29.6 | 63.6 | 103.9 | 50.9 | 38.5 | 62.5 | 44.3 | 16.9 | 45.8 |
| AFFX-ThrX-5_at | 10.7 | 11.6 | 4.4 | 4 | 10.5 | 10.8 | 13.6 | 4.4 | 6.9 | 4.4 | 6.4 | 15.6 | 10.2 | 7.1 |
| AFFX-ThrX-M_at | 20.2 | 24.1 | 44.3 | 25.8 | 27 | 42.3 | 46.6 | 40.6 | 28.8 | 32.6 | 38.2 | 47.3 | 23.2 | 12.9 |
| AFFX-ThrX-3_at | 7 | 9.5 | 7.8 | 7.7 | 5.6 | 8 | 10.1 | 7.2 | 8.3 | 5.4 | 12.7 | 10.3 | 8.2 | 8.2 |
| AFFX-TrpnX-5_at | 6.3 | 6.9 | 17.3 | 8.8 | 38.7 | 11.2 | 19.5 | 18.5 | 9.3 | 12.2 | 11.4 | 11.2 | 7 | 4.4 |
| AFFX-TrpnX-M_at | 7 | 5.5 | 6 | 5.8 | 8.7 | 9.6 | 10.1 | 5.3 | 3.8 | 7.1 | 6.2 | 7.6 | 4.2 | 6.5 |
| AFFX-TrpnX-3_at | 1.5 | 4.9 | 2.9 | 5.6 | 2.3 | 2.4 | 7.4 | 2 | 2.8 | 1.1 | 14.4 | 2.4 | 2.5 | 2.8 |
| AFFX-b-ActinMur/M12481_5_at | 17341.4 | 20464.3 | 16041.1 | 14948.8 | 16937.6 | 16905.7 | 18857.3 | 18513.8 | 19873.1 | 14590.4 | 18053.7 | 18020.8 | 16653.1 | 17434.7 |
| [illegible] | [illegible] | [illegible] | [illegible] | [illegible] | [illegible] | [illegible] | [illegible] | [illegible] | [illegible] | [illegible] | [illegible] | [illegible] | [illegible] | [illegible] |

Supplementary Table 1

Moggs et al. Phenotypic anchoring of gene expression changes during estrogen-induced uterine growth

[illegible]

Supplementary Table 1

Moggs et al. Phenotypic anchoring of gene expression changes during estrogen-induced uterine growth

[illegible]

Supplementary Table 1

Moggs et al. Phenotypic anchoring of gene expression changes during estrogen-induced uterine growth

Supplementary Table 1

Moggs et al. Phenotypic anchoring of gene expression changes during estrogen-induced uterine growth

Supplementary Table 1

Moggs et al. Phenotypic anchoring of gene expression changes during estrogen-induced uterine growth

[illegible]

Supplementary Table 1

Moggs et al. Phenotypic anchoring of gene expression changes during estrogen-induced uterine growth

Supplementary Table 1

Moggs et al. Phenotypic anchoring of gene expression changes during estrogen-induced uterine growth

[illegible]

Supplementary Table 1

Moggs et al. Phenotypic anchoring of gene expression changes during estrogen-induced uterine growth

[illegible]

Supplementary Table 1

[illegible]

Supplementary Table 1

Supplementary Table 1

Moggs et al. Phenotypic anchoring of gene expression changes during estrogen-induced uterine growth

[illegible]

Supplementary Table 1

Moggs et al. Phenotypic anchoring of gene expression changes during estrogen-induced uterine growth

Supplementary Table 1

[illegible]

Supplementary Table 1

Moggs et al. Phenotypic anchoring of gene expression changes during estrogen-induced uterine growth

[illegible]

Supplementary Table 1

| | | | | | | | | | | | | | | | |
|---|---|---|---|---|---|---|---|---|---|---|---|---|---|---|---|
| 99931_at | 1420 | 1305.3 | 1424.4 | 1308.2 | 1156.5 | 1290.3 | 1087.2 | 1430.7 | 1919.7 | 1383.3 | 1210.7 | 1090.7 | 1593.6 | 1495.9 | [illegible] |
| 99932_at | 172.8 | 72.7 | 84.4 | 74.5 | 83.5 | 84 | 81.6 | 76.1 | 77 | 86.3 | 72.9 | 59.4 | 75.7 | 83.9 | [illegible] |
| 99933_at | 712.5 | 768.5 | 791.2 | 589.2 | 554.1 | 605.2 | 661.8 | 610.9 | 882.7 | 723 | 697.8 | 518.2 | 784.9 | 735.7 | [illegible] |
| 99934_at | 700.2 | 622.6 | 692.8 | 622.1 | 555 | 633.1 | 606.2 | 656.2 | 679.6 | 569.7 | 597.4 | 740.6 | 813.9 | 707.3 | [illegible] |
| 99935_at | 2837 | 3270.3 | 2971.2 | 2671.8 | 2927 | 2520 | 2354.8 | 2348.6 | 2039.7 | 1565.5 | 1623 | 1678.5 | 1926.5 | 2182.9 | [illegible] |
| 99936_at | 797 | 669.7 | 822.1 | 654.3 | 624.4 | 556.4 | 532 | 697.3 | 630.8 | 506.2 | 276.2 | 289 | 330.5 | 527.9 | [illegible] |
| 99937_at | 98.1 | 89.2 | 68.6 | 106.1 | 103.1 | 94.8 | 72.4 | 43.2 | 1.2 | 12.3 | 29.5 | 8.7 | 10.1 | 10.5 | [illegible] |
| 99938_at | 356.6 | 419.9 | 332.4 | 328.3 | 376.6 | 406.7 | 328.7 | 350.7 | 290 | 292.4 | 336.8 | 327.4 | 259.4 | 262.2 | [illegible] |
| 99939_at | 328.1 | 405 | 447.4 | 363.9 | 402.8 | 500.6 | 430.2 | 419.4 | 386.7 | 363.9 | 435.1 | 460.3 | 345.6 | 518.4 | [illegible] |
| 99940_at | 819.1 | 767.8 | 912.3 | 780.7 | 770.7 | 906.8 | 696 | 937.2 | 1124.6 | 878.2 | 846.3 | 506.5 | 634.9 | 846.2 | [illegible] |
| 99941_at | 29.8 | 19.3 | 19.4 | 67.5 | 28.6 | 49.1 | 54.4 | 12 | 12 | 16.8 | 24.8 | 12.5 | 15 | 9.2 | [illegible] |
| 99942_s_at | 5630.5 | 6116.6 | 5264.3 | 5298.3 | 5314.7 | 5113.9 | 5163.5 | 4945 | 7327.3 | 5972.8 | 7085.4 | 5152.4 | 5604.8 | 5443.4 | [illegible] |
| 99944_at | 42.3 | 45.3 | 61.9 | 41.1 | 27.2 | 55.3 | 39.9 | 42.8 | 20.8 | 15.3 | 48.2 | 16.7 | 41.4 | 55 | [illegible] |
| 99945_at | 183.5 | 198 | 231.8 | 192.6 | 206.9 | 162.7 | 212.4 | 171.5 | 213.2 | 184.1 | 273.6 | 300.1 | 229.3 | 162.9 | [illegible] |
| 99947_at | 712.9 | 753.8 | 778.1 | 908.1 | 904 | 934 | 765.4 | 722 | 781.9 | 1177 | 1084 | 1064.4 | 1008.9 | 828.1 | [illegible] |
| 99948_at | 370.6 | 419.8 | 389.3 | 361.6 | 396.3 | 385.2 | 295.7 | 411.7 | 419.6 | 482.2 | 488.1 | 527.9 | 359.2 | 421.8 | [illegible] |
| 99949_at | 493.8 | 488.9 | 538.2 | 580.4 | 469.4 | 588.2 | 530.1 | 476.4 | 661.1 | 701.1 | 641.3 | 690.6 | 380.5 | 380.2 | [illegible] |
| 99950_at | 284 | 277.9 | 251 | 296.2 | 302.6 | 271.5 | 287.2 | 320.3 | 357.7 | 324 | 296.9 | 270.6 | 258 | 268.8 | [illegible] |
| 99951_at | 63.8 | 63 | 102.8 | 91.6 | 52.1 | 57.4 | 71.8 | 69.1 | 15.3 | 30.5 | 11.1 | 43 | 37.4 | 59.1 | [illegible] |
| 99952_at | 15.4 | 9 | 5.3 | 3.9 | 3.7 | 3.2 | 6.9 | 4.2 | 7.9 | 8.4 | 44.7 | 206.2 | 305 | 3.2 | [illegible] |
| 99953_at | 733.8 | 777.5 | 822.8 | 540.9 | 585.2 | 545.4 | 555.4 | 674.4 | 613.9 | 421.5 | 521 | 482.2 | 579.9 | 649.3 | [illegible] |
| 99954_at | 784.2 | 881.3 | 736 | 715.1 | 764.1 | 848.3 | 969.8 | 872.3 | 1089.1 | 1049.3 | 1324.2 | 1285.6 | 671.5 | 757 | [illegible] |
| 99955_at | 466.3 | 431.5 | 444.9 | 418.6 | 373 | 445.3 | 361.9 | 414.7 | 366 | 418.6 | 302.3 | 275.4 | 402 | 414.7 | [illegible] |
| 99956_at | 292.6 | 307.2 | 322.1 | 360.9 | 366.9 | 361.5 | 495.4 | 462.9 | 656.1 | 614.3 | 334.7 | 296.6 | 179.5 | 381.4 | [illegible] |
| 99957_at | 398.2 | 332.1 | 347.6 | 231.4 | 368.3 | 111.4 | 146.9 | 166.8 | 144.8 | 21.9 | 14.8 | 14.6 | 219 | 263 | [illegible] |
| 99958_at | 25.1 | 20.8 | 11.1 | 23.6 | 22.7 | 32.8 | 31.2 | 21 | 35.9 | 26.8 | 43.3 | 51.3 | 28.3 | 40.7 | [illegible] |
| 99959_at | 167.1 | 171.1 | 180.2 | 172.9 | 126.2 | 111.6 | 108.2 | 199.2 | 121.7 | 168.6 | 148.6 | 111 | 85 | 117.6 | [illegible] |
| 99960_at | 343.9 | 329.9 | 422.4 | 328.4 | 225.9 | 336.8 | 225.2 | 225.3 | 562 | 434.8 | 404.5 | 370.7 | 342.9 | 400.2 | [illegible] |
| 99961_s_at | 335.3 | 347.3 | 352.6 | 331.2 | 320.6 | 354.8 | 290 | 350.8 | 468 | 548.6 | 512.2 | 442.8 | 344.5 | 330.3 | [illegible] |
| 99962_at | 375.4 | 280.2 | 340.2 | 330.6 | 396 | 345.1 | 294.2 | 296.8 | 259.9 | 377.9 | 309.4 | 306.6 | 279.2 | 317.5 | [illegible] |
| 99963_at | 165 | 153.8 | 144 | 182.4 | 152.2 | 177.6 | 139.3 | 95.5 | 97.5 | 95.3 | 121.9 | 170.7 | 122.7 | 141.9 | [illegible] |
| 99964_at | 8.1 | 6.1 | 5.5 | 4.9 | 5.5 | 5.7 | 4.2 | 6.7 | 5.2 | 6.6 | 5.2 | 8.3 | 5.8 | 6.6 | [illegible] |
| 99965_at | 88.9 | 80.2 | 62.5 | 39.8 | 42.2 | 154 | 80.7 | 40.7 | 46.8 | 20.6 | 66.4 | 30.7 | 87.4 | 66.8 | [illegible] |
| 99966_at | 14.4 | 18.8 | 15.7 | 14.6 | 15.4 | 16.4 | 21.4 | 16.3 | 18 | 8.3 | 15.2 | 19 | 13.1 | 17.4 | [illegible] |
| 99970_at | 233.9 | 237.1 | 235.9 | 206.3 | 188.9 | 218.8 | 166.6 | 236.2 | 211.3 | 276.2 | 177.9 | 167.3 | 224.7 | 221 | [illegible] |
| 99972_at | 2.7 | 5.6 | 3.8 | 3.7 | 3.7 | 5.5 | 3.7 | 6.5 | 6.7 | 3.1 | 7.6 | 5.9 | 4 | 3.2 | [illegible] |
| 99973_s_at | 32.8 | 51.2 | 20.3 | 48.9 | 59.3 | 16.1 | 38.5 | 26.8 | 21.3 | 51.8 | 9.1 | 35 | 12.7 | 11.2 | [illegible] |
| 99974_at | 4.4 | 5.7 | 3.7 | 5.8 | 10.4 | 5 | 5.8 | 4.9 | 3.5 | 3.6 | 8.8 | 3.6 | 4.2 | 6.1 | [illegible] |
| 99975_at | 495 | 251.3 | 491 | 591.7 | 473.2 | 498.7 | 474.1 | 549.7 | 544.9 | 821.9 | 754.8 | 512.2 | 447.8 | 601.1 | [illegible] |
| 99977_at | 20.5 | 109.6 | 40.5 | 20.4 | 57.1 | 75.9 | 41.1 | 66.5 | 106.6 | 68.5 | 69.7 | 86.3 | 81.9 | 95.5 | [illegible] |
| [illegible] | [illegible] | [illegible] | [illegible] | [illegible] | [illegible] | [illegible] | [illegible] | [illegible] | [illegible] | [illegible] | [illegible] | [illegible] | [illegible] | [illegible] | [illegible] |

[illegible]

Supplementary Table 1

| | | | | | | | | | | | | | | | |
|---|---|---|---|---|---|---|---|---|---|---|---|---|---|---|---|
| 102670_at | 902.7 | 872.7 | 870.9 | 696.8 | 934.3 | 864.1 | 701.8 | 705.6 | 776.3 | 546 | 681.8 | 473 | 806.1 | 780.6 | [illegible] |
| 102671_at | 134.5 | 139.3 | 133.1 | 149.4 | 139 | 200.5 | 182.7 | 122.7 | 139.4 | 111.1 | 142.3 | 150.5 | 130.4 | 141.1 | [illegible] |
| 102672_g_at | 99.2 | 156.5 | 86.2 | 118.9 | 114.7 | 95.8 | 165 | 152.2 | 135.7 | 131.2 | 153.8 | 177.9 | 130.8 | 82.3 | [illegible] |
| 102673_at | 42.7 | 10.4 | 62.9 | 55.5 | 32.5 | 110.4 | 54.3 | 78.3 | 16.7 | 14.7 | 56.1 | 52.9 | 39.9 | 45 | [illegible] |
| 102674_at | 8.9 | 6.5 | 1.5 | 5.7 | 1.8 | 1.9 | 1.2 | 2 | 1.6 | 1.3 | 11 | 7.3 | 1.5 | 2.4 | [illegible] |
| 102675_at | 28.6 | 35 | 42.7 | 32.5 | 63.5 | 52.5 | 75.3 | 43.5 | 44.1 | 42.9 | 63.8 | 56.1 | 14.1 | 42.6 | [illegible] |
| 102676_at | 173.2 | 162.5 | 147.7 | 136.4 | 99.1 | 145.5 | 184.9 | 147.2 | 134.3 | 92.9 | 156.3 | 173.9 | 85.9 | 185 | [illegible] |
| 102677_at | 12.2 | 21.6 | 12.8 | 20.6 | 55.6 | 54.6 | 60.8 | 59.4 | 32 | 76.8 | 92.9 | 91.8 | 50.2 | 56.8 | [illegible] |
| 102678_at | 326.7 | 331.5 | 363.5 | 302.6 | 365.9 | 299 | 265.3 | 283.7 | 265.4 | 209.9 | 233.2 | 242 | 347.4 | 312.5 | [illegible] |
| 102679_at | 100.9 | 123.4 | 199.5 | 117.8 | 113.3 | 99.4 | 134.9 | 156.8 | 126.3 | 172.9 | 236.9 | 148.1 | 74 | 185.2 | [illegible] |
| 102680_g_at | 54.7 | 106 | 67.2 | 62.3 | 68.4 | 64.3 | 70 | 66.6 | 86.2 | 209.7 | 166.8 | 112.5 | 101.2 | 62.4 | [illegible] |
| 102681_at | 21.1 | 25.6 | 25.9 | 23.2 | 48.1 | 21.8 | 29 | 22.5 | 21.4 | 28.7 | 17 | 21.8 | 26.1 | 26.5 | [illegible] |
| 102682_at | 200.3 | 157.7 | 128.1 | 160.8 | 203.6 | 176.3 | 142.1 | 169.9 | 159.3 | 78.5 | 173.9 | 224.2 | 191.3 | 142 | [illegible] |
| 102683_at | 1532.6 | 1826.4 | 2053.7 | 1504.8 | 1663.4 | 1386 | 1789.5 | 1564.6 | 1847.4 | 1588.1 | 1439.8 | 1641.8 | 1511.5 | 2153.5 | [illegible] |
| 102684_at | 50.9 | 34 | 36.7 | 38.6 | 32 | 54.3 | 52.1 | 33.1 | 34.8 | 33.3 | 73.1 | 52.6 | 46.1 | 23.5 | [illegible] |
| 102685_at | 6.5 | 21.5 | 20.8 | 5.6 | 7.1 | 9.8 | 6.4 | 4.5 | 8.3 | 6.4 | 6.6 | 8.6 | 25.6 | 10.3 | [illegible] |
| 102686_at | 712.4 | 685.8 | 802.2 | 697.7 | 679.5 | 742.5 | 817.9 | 775.8 | 527.7 | 438.3 | 444.1 | 660.7 | 749.2 | 741.2 | [illegible] |
| 102687_at | 8 | 28.3 | 14.7 | 10.5 | 40.2 | 12.3 | 16.7 | 12.3 | 6.9 | 16.6 | 8.5 | 16.5 | 14.3 | 10.6 | [illegible] |
| 102688_f_at | 9.2 | 28.3 | 18.1 | 82.2 | 36.2 | 99.2 | 121.1 | 36.3 | 33.1 | 67.9 | 28.9 | 32.8 | 21.5 | 26.7 | [illegible] |
| 102689_at | 1079.4 | 1090.6 | 1194.5 | 819.6 | 940.5 | 905.1 | 1013.3 | 992.5 | 987.6 | 683.3 | 544.5 | 636.7 | 1224.7 | 831.9 | [illegible] |
| 102690_at | 27.7 | 42.1 | 41.9 | 26.9 | 33.9 | 25.9 | 38 | 35.9 | 25 | 20.1 | 41.9 | 44.4 | 33.4 | 25.8 | [illegible] |
| 102691_at | 511.6 | 363.5 | 532 | 406.1 | 397.4 | 547.7 | 598.2 | 527.3 | 388.4 | 432.6 | 475.5 | 455.8 | 430.3 | 598.1 | [illegible] |
| 102692_s_at | 54.3 | 25.2 | 56.2 | 27.7 | 59.9 | 45.8 | 23.8 | 44 | 25.4 | 23.7 | 72.8 | 41.1 | 41.1 | 49.1 | [illegible] |
| 102693_f_at | 13.9 | 15.4 | 18.1 | 11.9 | 12.6 | 17.9 | 18.5 | 175.9 | 237.7 | 308.4 | 158.8 | 29.1 | 22.2 | 21.2 | [illegible] |
| 102694_at | 2.4 | 1 | 1.5 | 12.6 | 1.8 | 2.3 | 1.7 | 1 | 1 | 0.7 | 1.4 | 1 | 2.5 | 1.2 | [illegible] |
| 102695_at | 127.9 | 138.4 | 169.3 | 97.4 | 143.2 | 171.9 | 175.7 | 133.9 | 119.7 | 85.9 | 150.8 | 105.9 | 79.8 | 115.3 | [illegible] |
| 102696_s_at | 199.1 | 214.3 | 205.2 | 265.5 | 229 | 185.4 | 254.2 | 302.1 | 358.1 | 406.2 | 377.8 | 213.1 | 187.2 | 186.1 | [illegible] |
| 102697_at | 479.5 | 653 | 582.8 | 510.1 | 470.5 | 596.5 | 598.6 | 553.5 | 787.2 | 608.3 | 852.4 | 663.5 | 449.5 | 531.9 | [illegible] |
| 102698_at | 415.7 | 379.4 | 543.5 | 437.5 | 337.9 | 305.9 | 311.1 | 545.8 | 702.3 | 744.8 | 367.2 | 269.6 | 294.1 | 458.7 | [illegible] |
| 102699_at | 70.3 | 73.6 | 53.2 | 50.7 | 50 | 48.7 | 60.3 | 58.4 | 79.8 | 47.5 | 50.3 | 53.1 | 66.3 | 58.2 | [illegible] |
| 102700_at | 2 | 11.9 | 6.8 | 3.3 | 16.2 | 8.1 | 3.8 | 5.7 | 14.7 | 18.8 | 9.4 | 4 | 2.3 | 11.3 | [illegible] |
| 102701_at | 127.5 | 92.5 | 30.2 | 160.4 | 114.4 | 114.4 | 169.7 | 156.5 | 102.7 | 126.1 | 213.3 | 191.9 | 80.8 | 135.6 | [illegible] |
| 102702_at | 241.5 | 188.4 | 234.9 | 223.9 | 222.7 | 212 | 189 | 222.8 | 215.4 | 226.2 | 230.8 | 223.2 | 207.6 | 263.6 | [illegible] |
| 102703_s_at | 4.5 | 13.6 | 6.4 | 16.5 | 12.3 | 3 | 6.7 | 4 | 5.3 | 4 | 17.8 | 7.7 | 19.9 | 24.7 | [illegible] |
| 102704_at | 11.1 | 20.6 | 2.7 | 10.5 | 1.9 | 25.9 | 13.8 | 25.2 | 29 | 18.5 | 46.2 | 40.6 | 80.3 | 67.3 | [illegible] |
| 102705_at | 3.7 | 4.4 | 4 | 4.9 | 3.6 | 4.6 | 6.1 | 2.1 | 3.4 | 3.9 | 6.4 | 4.8 | 5.1 | 5.2 | [illegible] |
| 102706_i_at | 21.1 | 24.6 | 33.4 | 27.9 | 25.9 | 13.8 | 12.2 | 8.1 | 5.3 | 16.3 | 18.9 | 18.9 | 6 | 20.8 | [illegible] |
| 102707_f_at | 89.7 | 114.2 | 71.7 | 63.2 | 113.9 | 102.7 | 116.2 | 117.5 | 97.5 | 62.9 | 112.2 | 121.3 | 66 | 133.7 | [illegible] |
| 102708_at | 13.3 | 46.2 | 16.3 | 24.7 | 32 | 21.8 | 14 | 14.8 | 21.2 | 10.4 | 11.2 | 16.7 | 21 | 12.7 | [illegible] |
| 102709_at | 9.1 | 24.8 | 58.9 | 51 | 19.2 | 40.4 | 47.9 | 52.5 | 25.8 | 10.2 | 46.5 | 34.7 | 30.5 | 27.1 | [illegible] |
| [illegible] | [illegible] | [illegible] | [illegible] | [illegible] | [illegible] | [illegible] | [illegible] | [illegible] | [illegible] | [illegible] | [illegible] | [illegible] | [illegible] | [illegible] | [illegible] |

Moggs et al. Phenotypic anchoring of gene expression changes during estrogen-induced uterine growth

| | | | | | | | | | | | | | | | |
|---|---|---|---|---|---|---|---|---|---|---|---|---|---|---|---|
| 103481_at | 359.7 | 394.1 | 447.6 | 474.2 | 491.2 | 443.9 | 449.2 | 384.1 | 134.1 | 154.2 | 218.5 | 219.3 | 229.9 | 316.2 | [illegible] |
| 103483_at | 215.6 | 285.4 | 241.9 | 228.1 | 227.8 | 217.8 | 192.5 | 162.5 | 204.3 | 189.3 | 190.4 | 214.8 | 168.8 | 248.8 | [illegible] |
| 103484_at | 5.4 | 9.6 | 10.5 | 6.6 | 14.3 | 1.8 | 10 | 8.5 | 17.9 | 27 | 14.3 | 11.6 | 7.1 | 9.6 | [illegible] |
| 103485_at | 505.5 | 497.9 | 590.7 | 547.3 | 360 | 425.7 | 379.5 | 534.5 | 449.3 | 362.4 | 336 | 323.7 | 290.9 | 448.6 | [illegible] |
| 103486_at | 11.3 | 15.7 | 20.9 | 13.2 | 11.3 | 9.5 | 24.7 | 49.5 | 25.5 | 22 | 14 | 45.8 | 17.7 | 62.8 | [illegible] |
| 103487_at | 809.9 | 820 | 680.3 | 759.7 | 649.8 | 575.6 | 551.6 | 810.8 | 698.5 | 512.8 | 519.7 | 625.6 | 571.4 | 645.7 | [illegible] |
| 103488_at | 365.5 | 437.1 | 421.9 | 333.8 | 375 | 405.8 | 442 | 369.8 | 328.8 | 254.8 | 340.6 | 304.2 | 320.7 | 400.5 | [illegible] |
| 103489_at | 135.8 | 182.8 | 93.8 | 121.9 | 94.7 | 141.7 | 124.4 | 131.5 | 127 | 72.1 | 76.2 | 79.7 | 95.1 | 138.7 | [illegible] |
| 103490_at | 208 | 140 | 59.3 | 69.4 | 143.1 | 52.8 | 16.2 | 118.6 | 117.7 | 19.4 | 4.4 | 4.7 | 6.7 | 76.9 | [illegible] |
| 103491_at | 405.9 | 335.1 | 351.5 | 349.6 | 428.1 | 364.2 | 389.7 | 445.5 | 252.6 | 429.5 | 471 | 474.7 | 456.6 | 381.2 | [illegible] |
| 103492_at | 453 | 352.1 | 454.2 | 530.9 | 385.1 | 417 | 343.2 | 355.9 | 273.3 | 142.4 | 192.2 | 207.6 | 256.6 | 302.9 | [illegible] |
| 103493_at | 67.6 | 92.8 | 25.8 | 49.8 | 45.7 | 72.7 | 40 | 81.8 | 103.2 | 69.3 | 45 | 19 | 73.3 | 57.8 | [illegible] |
| 103494_at | 280.6 | 319.5 | 301.1 | 291.9 | 266.7 | 236.8 | 237.8 | 243.4 | 233.5 | 108.1 | 158.3 | 382.9 | 836.2 | 612.3 | [illegible] |
| 103495_at | 8.7 | 20.6 | 13.3 | 17.6 | 14.3 | 16.8 | 16.8 | 16.6 | 3.8 | 14 | 27.2 | 21.8 | 13.1 | 5.8 | [illegible] |
| 103496_at | 40 | 47 | 29.7 | 34.3 | 44.5 | 15.4 | 42.1 | 22.3 | 20.4 | 44.1 | 42.1 | 35.5 | 57.8 | 79.3 | [illegible] |
| 103497_at | 140.2 | 150.3 | 126.4 | 139.5 | 145.9 | 106.7 | 150.6 | 139.5 | 172.9 | 226.1 | 205.8 | 164.6 | 138.8 | 153 | [illegible] |
| 103498_at | 150.9 | 125.7 | 183.4 | 182.9 | 151 | 152.3 | 258.4 | 226.9 | 215 | 159.7 | 179.9 | 238.2 | 201.9 | 201.4 | [illegible] |
| 103499_at | 315 | 263.7 | 339 | 241.9 | 240.8 | 220.9 | 236.4 | 187 | 245.5 | 192.4 | 126.8 | 65.8 | 233.1 | 236.4 | [illegible] |
| 103500_at | 631 | 544.5 | 560.4 | 576.7 | 588 | 546.1 | 532.3 | 499.6 | 320.2 | 441.6 | 382.8 | 339 | 447.3 | 637.5 | [illegible] |
| 103501_at | 1679 | 1590.8 | 1158.8 | 1154.1 | 1139.6 | 958.6 | 1389.6 | 1686.6 | 1563.7 | 1187.4 | 995.4 | 723.7 | 774.2 | 1440.5 | [illegible] |
| 103502_at | 10 | 25.9 | 57.4 | 66.8 | 66.1 | 52.5 | 53 | 15.7 | 47.1 | 48.4 | 47 | 133.8 | 71.3 | 12.8 | [illegible] |
| 103503_at | 73.2 | 43.4 | 95 | 70.5 | 115.4 | 88.3 | 43 | 82.7 | 50.7 | 69.8 | 94.7 | 114.7 | 111.6 | 84.3 | [illegible] |
| 103504_at | 1645.3 | 1357.7 | 1438.1 | 1531.9 | 1466.5 | 1355.1 | 1295.8 | 1466.5 | 965.2 | 451.4 | 636.6 | 761.3 | 903.7 | 1129.5 | [illegible] |
| 103506_f_at | 148.4 | 119.5 | 117 | 147.7 | 122.7 | 145.4 | 117.3 | 175 | 364.3 | 321.5 | 220.6 | 180.6 | 155.3 | 152.8 | [illegible] |
| 103507_at | 22.5 | 37.5 | 27.6 | 17.7 | 21.4 | 62 | 59.6 | 25.8 | 70.4 | 14.2 | 18.2 | 21 | 136.7 | 142.3 | [illegible] |
| 103508_at | 76.9 | 43.1 | 65.3 | 40.2 | 64.9 | 76.6 | 58.7 | 61 | 52.5 | 58.5 | 37.7 | 71.7 | 83.7 | 51.4 | [illegible] |
| 103509_at | 2.2 | 4.5 | 3.6 | 3.2 | 7 | 4.3 | 7 | 4.8 | 8 | 7.5 | 7 | 3.6 | 3.2 | 7.3 | [illegible] |
| 103510_at | 83 | 101 | 58.4 | 57.8 | 54.6 | 125.5 | 24.7 | 68.8 | 94.5 | 105.1 | 156.3 | 60.3 | 76 | 79.1 | [illegible] |
| 103511_at | 313.8 | 225.8 | 198.1 | 278.4 | 225.2 | 206.5 | 284.6 | 635.7 | 958.9 | 419.2 | 551.5 | 335.8 | 208 | 200.7 | [illegible] |
| 103512_at | 423.9 | 410.8 | 306.7 | 346.9 | 280.1 | 381.2 | 432.5 | 446.8 | 419.4 | 381 | 550.1 | 486.5 | 378.8 | 390.7 | [illegible] |
| 103513_at | 119.8 | 116.4 | 126.4 | 127.4 | 110 | 157.8 | 160.2 | 138.2 | 158.2 | 104 | 147.2 | 93.1 | 101.1 | 95.7 | [illegible] |
| 103514_at | 437.3 | 333.3 | 389.1 | 367.4 | 444.7 | 320 | 239.4 | 210.7 | 195.7 | 220 | 211.3 | 219.9 | 432.2 | 417.6 | [illegible] |
| 103515_at | 1109.2 | 1217.8 | 1091.4 | 1078.5 | 995.4 | 1123.9 | 1156.8 | 1159.8 | 912.7 | 936.1 | 982.8 | 1002.9 | 1022.6 | 1092.5 | [illegible] |
| 103516_at | 193.2 | 201 | 226.1 | 186.4 | 140.9 | 155.8 | 143.8 | 168.5 | 220.7 | 177.9 | 147.4 | 163.6 | 277.8 | 258 | [illegible] |
| 103517_at | 55.1 | 47.3 | 38.8 | 45.5 | 41.5 | 38.1 | 43.3 | 56.4 | 53.9 | 62.9 | 23.5 | 11 | 75.2 | 66.8 | [illegible] |
| 103518_at | 124.7 | 141.7 | 112.8 | 128.4 | 123.1 | 123.1 | 128.1 | 176 | 201.2 | 188.8 | 170.8 | 106.5 | 149.2 | 121.8 | [illegible] |
| 103519_at | 29.6 | 7.2 | 13.6 | 7.6 | 16.8 | 10.2 | 20.9 | 6.1 | 7.4 | 7.9 | 32.5 | 29.4 | 11.4 | 10.5 | [illegible] |
| 103520_at | 342.9 | 444.2 | 332.5 | 391.9 | 405 | 341.6 | 373.8 | 1637.4 | 1805 | 870.8 | 479.5 | 219.6 | 204.8 | 230.7 | [illegible] |
| 103521_r_at | 18.6 | 26.7 | 21.3 | 18.6 | 20.8 | 22.7 | 20.5 | 19.8 | 20.7 | 16.9 | 49.6 | 21.9 | 27.7 | 22.7 | [illegible] |
| 103522_at | 29.8 | 16 | 20.8 | 20.6 | 25.2 | 47.1 | 50.3 | 28.9 | 5.2 | 11.9 | 15.3 | 30.5 | 10.3 | 18.7 | [illegible] |
| [illegible] | [illegible] | [illegible] | [illegible] | [illegible] | [illegible] | [illegible] | [illegible] | [illegible] | [illegible] | [illegible] | [illegible] | [illegible] | [illegible] | [illegible] | [illegible] |

Supplementary Table 1

Moggs et al. Phenotypic anchoring of gene expression changes during estrogen-induced uterine growth

[illegible]

Supplementary Table 1

Moggs et al. Phenotypic anchoring of gene expression changes during estrogen-induced uterine growth

[illegible]

Supplementary Table 1

Moggs et al. Phenotypic anchoring of gene expression changes during estrogen-induced uterine growth

[illegible]

Supplementary Table 1

Moggs et al. Phenotypic anchoring of gene expression changes during estrogen-induced uterine growth

[illegible]

Supplementary Table 1

Moggs et al. Phenotypic anchoring of gene expression changes during estrogen-induced uterine growth

| | | | | | | | | | | | | | | | |
|---|---|---|---|---|---|---|---|---|---|---|---|---|---|---|---|
| 94706_x_at | 574.5 | 494.5 | 532.2 | 497.3 | 562.6 | 544.9 | 669.2 | 516.6 | 516.3 | 414.6 | 550.8 | 681.9 | 537.9 | 637.2 | [illegible] |
| 94707_x_at | 28.4 | 16.6 | 20.9 | 26.5 | 20.5 | 23.2 | 19.1 | 17 | 23.6 | 17.2 | 15.4 | 27 | 19.3 | 20 | [illegible] |
| 94708_at | 73.7 | 74.7 | 69.2 | 54.6 | 83.2 | 82.1 | 86.6 | 71.1 | 76.3 | 59.9 | 86.8 | 98.8 | 57.6 | 48.5 | [illegible] |
| 94709_at | 92.9 | 124.1 | 110.3 | 84.4 | 87.7 | 158.2 | 126.3 | 144.9 | 114.1 | 100.7 | 117.1 | 150 | 113.5 | 124.7 | [illegible] |
| 94710_g_at | 7.2 | 8.1 | 6.7 | 7.9 | 4.6 | 24.3 | 4.9 | 3.3 | 7 | 4.3 | 4.2 | 9.4 | 8.7 | 5.1 | [illegible] |
| 94711_at | 75.8 | 84.2 | 73.4 | 59.7 | 22.2 | 87.1 | 17.3 | 65.7 | 52.9 | 65.5 | 92.3 | 52.2 | 83.9 | 76.2 | [illegible] |
| 94712_at | 376.4 | 320.6 | 286.8 | 337.8 | 305 | 306.4 | 192.1 | 191.2 | 122.8 | 107.1 | 110.7 | 101 | 142.1 | 272.4 | [illegible] |
| 94713_at | 160.3 | 170.4 | 195.1 | 123 | 195.1 | 205.3 | 263.3 | 245.8 | 166.7 | 167.3 | 113.5 | 206.7 | 211.8 | 162.1 | [illegible] |
| 94714_at | 15.9 | 5.1 | 3.8 | 2.7 | 2.3 | 11.1 | 14 | 5.6 | 4.8 | 3 | 2.4 | 23.2 | 3.5 | 15.4 | [illegible] |
| 94715_at | 41.1 | 23.8 | 39.9 | 38.7 | 30.2 | 43 | 38.5 | 38.1 | 45.5 | 23 | 42.7 | 33.1 | 26.9 | 35.5 | [illegible] |
| 94716_f_at | 83.1 | 51 | 52.6 | 66.3 | 90 | 68.8 | 95.1 | 319.1 | 873.8 | 864.6 | 496 | 222.4 | 173 | 94.7 | [illegible] |
| 94717_f_at | 55.3 | 29.4 | 21.6 | 27.5 | 27.1 | 34.7 | 64.1 | 43.9 | 41.7 | 34.9 | 70.7 | 35.9 | 30.5 | 40.2 | [illegible] |
| 94718_at | 1.7 | 2.5 | 2.4 | 2 | 2.1 | 2.7 | 2.7 | 2.1 | 1.8 | 2 | 2.3 | 2.4 | 2.7 | 2.3 | [illegible] |
| 94719_at | 4.9 | 8.6 | 10.1 | 7.7 | 5.9 | 9.3 | 8.1 | 8.9 | 12.3 | 2.2 | 6 | 2.8 | 2.4 | 2.7 | [illegible] |
| 94720_at | 285.7 | 318.2 | 270.5 | 271.8 | 277.1 | 245 | 263.8 | 297.2 | 177.5 | 296.3 | 208.3 | 223.6 | 211.3 | 252.6 | [illegible] |
| 94721_at | 6.5 | 26.3 | 9 | 15.8 | 16.5 | 13.8 | 11.3 | 8 | 22.8 | 7.1 | 40 | 43.3 | 5.8 | 8.6 | [illegible] |
| 94722_f_at | 17.4 | 22.6 | 14.8 | 17.7 | 17.3 | 19.3 | 23.9 | 22.8 | 20.8 | 11.8 | 25.5 | 17.9 | 14 | 20.2 | [illegible] |
| 94723_at | 23.1 | 10.8 | 21.1 | 10.2 | 23.1 | 20 | 40.5 | 40.1 | 29.4 | 8 | 48.8 | 8.6 | 7 | 7.7 | [illegible] |
| 94724_at | 1.9 | 5.6 | 5.1 | 5.6 | 2.4 | 3.4 | 5.9 | 1.3 | 2 | 1.8 | 4.4 | 2.8 | 1.8 | 24.7 | [illegible] |
| 94725_f_at | 214.7 | 260.1 | 336.9 | 221 | 254.2 | 261.3 | 304.1 | 328.3 | 248.7 | 194.1 | 285 | 312.7 | 271.3 | 323.5 | [illegible] |
| 94726_at | 8.2 | 12.1 | 13.6 | 11 | 7.4 | 14.8 | 18.1 | 11.1 | 17 | 11.2 | 14.5 | 13.9 | 12.7 | 14.1 | [illegible] |
| 94727_f_at | 36.6 | 85.2 | 66.6 | 45.5 | 43.8 | 78.2 | 23.5 | 89.8 | 50.6 | 57.8 | 51 | 29.3 | 44.9 | 50.1 | [illegible] |
| 94728_f_at | 10.3 | 20.6 | 19.7 | 25.8 | 22.5 | 18.2 | 27.6 | 7.5 | 15.9 | 7.8 | 17.9 | 17 | 15.3 | 20.5 | [illegible] |
| 94729_r_at | 14.7 | 18 | 41.6 | 20.3 | 25.9 | 17.5 | 22.5 | 31.9 | 21.6 | 19.1 | 23.1 | 27.8 | 20.8 | 24.5 | [illegible] |
| 94730_at | 11.1 | 5.4 | 5.3 | 2.7 | 2.2 | 2.8 | 2.2 | 3.7 | 1.3 | 3.1 | 3.4 | 4.4 | 6.3 | 1.5 | [illegible] |
| 94731_at | 87.8 | 82.9 | 87 | 55.5 | 73.9 | 81.8 | 82.6 | 75.1 | 63.1 | 29 | 71.5 | 58.3 | 62.6 | 94.4 | [illegible] |
| 94732_at | 13.5 | 17.8 | 10.6 | 9.4 | 6.5 | 23.9 | 20.5 | 15.8 | 23 | 7.6 | 29.9 | 30.1 | 35.4 | 14 | [illegible] |
| 94733_at | 111.1 | 83.4 | 76.7 | 124.8 | 107 | 114.9 | 66 | 139.5 | 85.3 | 77.4 | 27 | 154.7 | 53.7 | 119.2 | [illegible] |
| 94734_at | 9.2 | 22 | 11.2 | 12.1 | 13.1 | 9.9 | 7.6 | 10.2 | 24.6 | 9.6 | 15.5 | 14.1 | 41.1 | 25.9 | [illegible] |
| 94735_x_at | 140.2 | 126.3 | 167.2 | 107.2 | 130.3 | 162.5 | 178.4 | 148.7 | 83.3 | 97.6 | 148.6 | 178.8 | 131.1 | 129.2 | [illegible] |
| 94736_at | 118.2 | 112.1 | 128.1 | 112.6 | 96.2 | 100.6 | 125.4 | 126.6 | 48.5 | 77 | 97.5 | 79.4 | 87.6 | 101.1 | [illegible] |
| 94737_at | 12 | 21.6 | 18 | 14.7 | 16.7 | 20.8 | 17.3 | 12.8 | 15.9 | 18.3 | 21 | 24.8 | 23.6 | 14.3 | [illegible] |
| 94738_x_at | 0.7 | 1.1 | 1.4 | 1.2 | 0.8 | 1.1 | 1.2 | 1.9 | 1.7 | 1.3 | 1.3 | 1.2 | 1.4 | 0.8 | [illegible] |
| 94739_at | 150.7 | 120.3 | 131.8 | 124.3 | 115.4 | 135.4 | 97.8 | 103.2 | 124.6 | 92.2 | 98.1 | 78.5 | 90.6 | 98.1 | [illegible] |
| 94740_g_at | 42.4 | 48.9 | 53.8 | 45.3 | 30.4 | 18 | 24.5 | 17.3 | 8.1 | 8.2 | 7.9 | 5.5 | 9.4 | 8.2 | [illegible] |
| 94741_at | 10.9 | 27.1 | 5.7 | 23 | 23.6 | 11.4 | 6.9 | 10.2 | 7.5 | 5.9 | 27.5 | 6.5 | 19.9 | 8.2 | [illegible] |
| 94742_f_at | 542.1 | 587.5 | 582.3 | 597.1 | 587.1 | 522 | 581.3 | 523.1 | 323.1 | 451.6 | 243.8 | 384.5 | 1150.6 | 825.8 | [illegible] |
| 94744_at | 131.2 | 127.6 | 214.9 | 114.9 | 148.6 | 204.8 | 161.1 | 120.4 | 141.2 | 122.2 | 105 | 108 | 160 | 192.7 | [illegible] |
| 94745_f_at | 47.7 | 58.9 | 25.3 | 49.9 | 34.2 | 63.5 | 62.9 | 59.4 | 70.8 | 84.7 | 78.2 | 71.7 | 56.7 | 47.9 | [illegible] |
| 94746_at | 62.8 | 12 | 10.9 | 26.1 | 17.8 | 11.7 | 26.7 | 29.9 | 29.5 | 15.8 | 46.4 | 28.9 | 41.3 | 27.1 | [illegible] |
| [illegible] | [illegible] | [illegible] | [illegible] | [illegible] | [illegible] | [illegible] | [illegible] | [illegible] | [illegible] | [illegible] | [illegible] | [illegible] | [illegible] | [illegible] | [illegible] |

Supplementary Table 1

Moggs et al. Phenotypic anchoring of gene expression changes during estrogen-induced uterine growth

Supplementary Table 1

Moggs et al. Phenotypic anchoring of gene expression changes during estrogen-induced uterine growth

Supplementary Table 1

Moggs et al. Phenotypic anchoring of gene expression changes during estrogen-induced uterine growth

Supplementary Table 1

Moggs et al. Phenotypic anchoring of gene expression changes during estrogen-induced uterine growth

| | | | | | | | | | | | | | | | | | | |
|---|---|---|---|---|---|---|---|---|---|---|---|---|---|---|---|---|---|---|
| 101730_at | 221.5 | 137.4 | 122.2 | 161.1 | 213.7 | 208.5 | 151.8 | 168.8 | 112.3 | 151.2 | 78.5 | 152.4 | 187 | 112.6 | 95.8 | 133.2 | 153.3 | 127.1 | [illegible] |
| 101731_at | 1.1 | 3.2 | 5.2 | 9.9 | 7.6 | 10.5 | 3.6 | 2.7 | 4.9 | 3.4 | 7.9 | 10.2 | 2.7 | 4.5 | 5.9 | 6.5 | 5 | 20.3 | [illegible] |
| 101732_at | 162.8 | 139.8 | 212.7 | 151.3 | 162.9 | 185.9 | 180.3 | 193.1 | 131.1 | 145.3 | 160.4 | 171.4 | 185.9 | 204.4 | 132.2 | 181.2 | 141.4 | 161.2 | [illegible] |
| 101733_at | 55.8 | 72.6 | 45.8 | 63.8 | 56 | 35.4 | 81.9 | 80.7 | 41.1 | 46.5 | 70.1 | 67.1 | 72.3 | 76.6 | 43.3 | 87.2 | 53.4 | 36.4 | [illegible] |
| 101734_at | 97.3 | 161 | 90.5 | 91.6 | 199.9 | 49.4 | 167.1 | 102.1 | 56.9 | 108.1 | 43.1 | 155.6 | 86.6 | 97.7 | 197.6 | 71.9 | 193.4 | 182.5 | [illegible] |
| 101735_f_at | 120.2 | 90.2 | 85.1 | 93.9 | 52.1 | 102.4 | 89.3 | 80.3 | 98.3 | 24.1 | 89.9 | 71.9 | 82 | 93.8 | 103.5 | 115 | 106.8 | 98.9 | [illegible] |
| 101736_at | 17.6 | 18.3 | 11.1 | 19.4 | 15.6 | 29.3 | 10.9 | 90.2 | 17.4 | 32.4 | 14.1 | 26.1 | 9.3 | 21.3 | 31.3 | 17.6 | 22.1 | 30.6 | [illegible] |
| 101737_at | 42.9 | 47.1 | 31.9 | 42.7 | 26.7 | 46.2 | 57 | 20.8 | 56.2 | 42.7 | 53.9 | 53.7 | 33.7 | 9.2 | 87 | 46.7 | 49.8 | 27 | [illegible] |
| 101738_at | 386.5 | 457.1 | 442.9 | 405.8 | 417.9 | 419.6 | 473.4 | 496 | 324.1 | 411.3 | 432.1 | 504.5 | 302.2 | 429.7 | 459.6 | 537.5 | 425.4 | 434.1 | [illegible] |
| 101739_at | 14.7 | 27.2 | 27.1 | 24.4 | 20.1 | 29.1 | 21.5 | 20.9 | 9.7 | 10.6 | 12.6 | 13.2 | 16.2 | 22.7 | 15.8 | 14.8 | 29.4 | 32.1 | [illegible] |
| 101740_at | 23.7 | 56.4 | 39.1 | 26 | 33.7 | 48.2 | 53.1 | 53.2 | 39.7 | 44.4 | 33.7 | 31.1 | 20.9 | 35.3 | 93.8 | 27.6 | 58.2 | 128.5 | [illegible] |
| 101741_at | 1832.7 | 1234.5 | 1694.2 | 1751.1 | 2411.2 | 1518.2 | 2391.1 | 2152.1 | 1636.4 | 1947.8 | 2396.5 | 2541.2 | 1985.8 | 2206 | 924.4 | 965.5 | 1455.1 | 1000.3 | [illegible] |
| 101742_at | 17 | 14.1 | 6.4 | 21.2 | 14.3 | 27.3 | 17.6 | 2.7 | 3.5 | 10 | 21.7 | 3.1 | 15.3 | 11.2 | 5.8 | 16.7 | 23 | 21.6 | [illegible] |
| 101743_f_at | 96.8 | 36.8 | 86.7 | 90.8 | 123.4 | 65.9 | 62.3 | 128.6 | 81.1 | 99 | 51.6 | 95.9 | 82.9 | 59.7 | 125.9 | 139 | 106 | 118.8 | [illegible] |
| 101744_i_at | 1.1 | 2.8 | 1.9 | 3.3 | 3.9 | 2.7 | 14.5 | 3 | 2.3 | 2.2 | 1.9 | 2.3 | 8.9 | 3 | 5.1 | 5.8 | 3.9 | 6.1 | [illegible] |
| 101745_f_at | 4.4 | 15 | 5.3 | 7 | 5 | 7.3 | 7 | 6.9 | 6 | 6.6 | 8.3 | 7.7 | 9.1 | 7.8 | 49 | 10.7 | 9.7 | 30.2 | [illegible] |
| 101746_i_at | 6.3 | 0.4 | 1.4 | 3.5 | 2.6 | 1.6 | 1.1 | 0.3 | 0.6 | 0.5 | 8.7 | 6.4 | 1 | 1.4 | 0.8 | 1.6 | 2.2 | 2.2 | [illegible] |
| 101747_f_at | 6.1 | 12.8 | 9.9 | 7.9 | 6.1 | 12.3 | 12.7 | 7.7 | 25.3 | 10.3 | 14 | 10.1 | 8.3 | 4.8 | 9.4 | 12.8 | 17.5 | 8.6 | [illegible] |
| 101748_at | 288.7 | 371.4 | 277.6 | 264.4 | 149.6 | 329.2 | 352 | 902.5 | 1197.9 | 660.7 | 752.7 | 272.7 | 287.4 | 268.3 | 380.3 | 222.4 | 225 | 233.1 | [illegible] |
| 101749_at | 3.6 | 17 | 11.4 | 3.8 | 6.1 | 15.6 | 15 | 8.4 | 1.6 | 1.2 | 24.6 | 5.2 | 3.8 | 14.7 | 38.3 | 33.2 | 8.3 | 26.9 | [illegible] |
| 101751_f_at | 72.1 | 85.6 | 75.7 | 50.2 | 24.2 | 53.1 | 75.1 | 77.5 | 31.3 | 47.7 | 59.3 | 77.6 | 54.3 | 25.7 | 69.9 | 23 | 117 | 87 | [illegible] |
| 101752_f_at | 9.8 | 4.6 | 6.2 | 5.3 | 8.6 | 10.1 | 7 | 9.8 | 7.2 | 4.9 | 9.5 | 4.2 | 6.3 | 5.8 | 15.3 | 3.7 | 4.2 | 6.7 | [illegible] |
| 101753_s_at | 349 | 468.6 | 402.5 | 322 | 348.6 | 491 | 600.5 | 249 | 345.5 | 443.6 | 340.1 | 180.2 | 563.4 | 1101.2 | 311 | 296.2 | 381.6 | 470.1 | [illegible] |
| 101754_f_at | 1287.7 | 1253.6 | 1402.4 | 1113.2 | 1176.6 | 1319 | 1199.7 | 1103.3 | 1488.4 | 2963.4 | 2001.3 | 5658.2 | 5634.2 | 2787.5 | 1320.2 | 1481.6 | 1485.5 | 1690.8 | [illegible] |
| 101755_f_at | 308.6 | 292.6 | 577.9 | 300.8 | 388.3 | 306.4 | 441.7 | 446.9 | 432 | 811.1 | 796.9 | 1836 | 1771.1 | 1098.8 | 292.2 | 464.1 | 388.5 | 722.4 | [illegible] |
| 101756_f_at | 142.8 | 137.1 | 204.9 | 131.3 | 110.6 | 193.8 | 137 | 141.2 | 101.5 | 191.1 | 182.7 | 209 | 279.1 | 192.9 | 202.7 | 204.3 | 249.3 | 146.1 | [illegible] |
| 101757_at | 635.5 | 631.4 | 619.8 | 508.1 | 526.4 | 387.5 | 498.5 | 821.6 | 1172.2 | 768.1 | 622 | 480.5 | 438.8 | 590.5 | 320.3 | 439 | 351.8 | 575.2 | [illegible] |
| 101758_at | 16.3 | 19.9 | 22.4 | 13.7 | 15.2 | 26.8 | 24.9 | 17.3 | 26.8 | 14.1 | 42.4 | 17.5 | 20.8 | 24.7 | 25.3 | 28.5 | 23 | 34.2 | [illegible] |
| 101759_at | 27.4 | 30.5 | 46.6 | 67.6 | 59.7 | 48.4 | 53.3 | 49.4 | 41.9 | 51.2 | 54.8 | 44 | 48.1 | 61.5 | 54.1 | 35.2 | 52.1 | 70.6 | [illegible] |
| 101760_at | 14.7 | 21 | 17 | 15 | 17.5 | 12.1 | 30.2 | 36.8 | 60.6 | 54.7 | 53.3 | 141.1 | 295.4 | 176 | 14.6 | 19.4 | 19.3 | 20 | [illegible] |
| 101761_f_at | 35.3 | 44.2 | 34.3 | 38.7 | 38.8 | 37.8 | 40 | 30.6 | 37.9 | 285.8 | 371.6 | 3860.2 | 2315 | 737.8 | 60.8 | 61 | 42.5 | 63.8 | [illegible] |
| 101762_at | 35.7 | 64.5 | 95 | 56.3 | 38.3 | 96.5 | 57.5 | 75.9 | 71.7 | 58 | 101.6 | 22 | 80.9 | 48.6 | 149.1 | 99.7 | 84.9 | 151.4 | [illegible] |
| 101763_at | 900.1 | 1035.2 | 1054.9 | 941.7 | 880.5 | 879.2 | 1063.6 | 993.6 | 866.9 | 885.7 | 986.8 | 910.8 | 782 | 972.2 | 1423.6 | 1308.3 | 1211.9 | 1454.7 | [illegible] |
| 101764_at | 224.8 | 252.8 | 294.8 | 241.9 | 285.1 | 234.8 | 271.8 | 283.1 | 348.3 | 349.7 | 230.5 | 258.6 | 332.8 | 326.7 | 205 | 274.4 | 230.2 | 214.5 | [illegible] |
| 101765_at | 44.5 | 49.1 | 47.9 | 23.9 | 30.6 | 50.2 | 47.9 | 48.3 | 51.9 | 39.1 | 48.2 | 55.9 | 40.3 | 49.7 | 58.2 | 63.5 | 49 | 62.6 | [illegible] |
| 101768_at | 5.6 | 18.1 | 6.3 | 11.5 | 7.4 | 8.2 | 9.3 | 32.9 | 7.9 | 15.2 | 31.1 | 17.9 | 4.4 | 24 | 49.1 | 9.5 | 10.3 | 16.3 | [illegible] |
| 101769_at | 141.2 | 126.3 | 156.8 | 156.4 | 185.6 | 191.7 | 205.1 | 85.1 | 126.1 | 96.2 | 282 | 239.2 | 189.6 | 109.2 | 283.3 | 261.4 | 256.1 | 199.7 | [illegible] |
| 101770_i_at | 1.5 | 2.6 | 4.4 | 3.9 | 2.4 | 4.6 | 5 | 5.2 | 1.6 | 3.4 | 4.8 | 4.2 | 2.6 | 4.7 | 4.6 | 3.3 | 5.6 | 5.9 | [illegible] |
| 101771_r_at | 893.3 | 997.1 | 1208.6 | 763.5 | 1030.7 | 1049.9 | 879 | 843.4 | 785 | 551.4 | 678.3 | 994.5 | 754.3 | 1701.3 | 961.3 | 975.7 | 621.6 | 992.8 | [illegible] |
| 101772_r_at | 157.8 | 184.6 | 225.1 | 151.8 | 128 | 109.7 | 163.1 | 185 | 166.9 | 178.1 | 220.6 | 91.6 | 142.9 | 202.7 | 187.8 | 139 | 104.4 | 321.6 | [illegible] |
| [illegible] | [illegible] | [illegible] | [illegible] | [illegible] | [illegible] | [illegible] | [illegible] | [illegible] | [illegible] | [illegible] | [illegible] | [illegible] | [illegible] | [illegible] | [illegible] | [illegible] | [illegible] | [illegible] | [illegible] |

Supplementary Table 1

Moggs et al. Phenotypic anchoring of gene expression changes during estrogen-induced uterine growth

[illegible]

Supplementary Table 1

Moggs et al. Phenotypic anchoring of gene expression changes during estrogen-induced uterine growth

[illegible]

Moggs et al. Phenotypic anchoring of gene expression changes during estrogen-induced uterine growth

[illegible]

Supplementary Table 1

[illegible]

Moggs et al. Phenotypic anchoring of gene expression changes during estrogen-induced uterine growth

[illegible]

Supplementary Table 1

Moggs et al. Phenotypic anchoring of gene expression changes during estrogen-induced uterine growth

[illegible]

Supplementary Table 1

| | | | | | | | | | | | | | | | | |
|---|---|---|---|---|---|---|---|---|---|---|---|---|---|---|---|---|
| 161611_f_at | 625.2 | 386.6 | 540.4 | 626.2 | 808.6 | 635.1 | 697.9 | 597.5 | 516.4 | 622.8 | 416.5 | 425 | 385.5 | 360.5 |
| 161612_f_at | 108.7 | 108.2 | 130.6 | 110.8 | 111.5 | 136.2 | 149.3 | 107.9 | 52.6 | 101.5 | 162.5 | 145 | 96.4 | 90.4 |
| 161613_at | 307.1 | 320.4 | 283.6 | 287.3 | 310.7 | 269.6 | 293.9 | 280.7 | 283.9 | 302.3 | 289.8 | 304.4 | 303.6 | 238.8 |
| 161614_r_at | 106.5 | 227.3 | 154 | 81.9 | 118.2 | 113 | 207.7 | 283.1 | 129.4 | 106.1 | 97.3 | 174.3 | 184.1 | 211.1 |
| 161615_f_at | 168.4 | 217.9 | 150.2 | 172.4 | 161.8 | 157.2 | 231.8 | 242.7 | 163.6 | 200.1 | 193.5 | 184.4 | 206.1 | 195.7 |
| 161616_f_at | 417.4 | 291.4 | 273.5 | 370.8 | 435.9 | 315.8 | 272.5 | 282.3 | 194.5 | 273.8 | 173.8 | 201.9 | 187.2 | 262.2 |
| 161617_f_at | 16.3 | 24.1 | 26.2 | 25.4 | 27.5 | 21 | 27 | 22.3 | 14 | 17.8 | 22.5 | 20.6 | 28.7 | 24 |
| 161618_r_at | 300 | 384.8 | 366.1 | 251.3 | 264.5 | 286.6 | 427.7 | 304.5 | 270.3 | 251.9 | 354.9 | 441.6 | 248.4 | 284 |
| 161619_f_at | 96.6 | 86.7 | 114.4 | 121.3 | 86.7 | 81.9 | 126.3 | 112.5 | 88.8 | 80 | 62.6 | 29.8 | 49.9 | 77.2 |
| 161620_f_at | 29.2 | 49.2 | 47.6 | 38.3 | 30.1 | 34.3 | 50.1 | 39.2 | 45.2 | 35.7 | 33.1 | 52.1 | 30.3 | 32.6 |
| 161621_r_at | 47 | 50.5 | 60.6 | 39.1 | 51.2 | 121 | 127.5 | 89.1 | 67.6 | 40.8 | 113.2 | 116.1 | 60.3 | 57.2 |
| 161622_f_at | 4 | 6.1 | 4 | 2.4 | 11.2 | 5.9 | 2.7 | 2.3 | 1.5 | 5.1 | 1.7 | 7.2 | 24.2 | 7.3 |
| 161623_at | 14.8 | 6.8 | 8 | 3.8 | 6.2 | 19.6 | 5.6 | 8.9 | 3.9 | 17.2 | 5.3 | 1.6 | 5 | 4.8 |
| 161624_r_at | 25.7 | 22.5 | 22.2 | 33.2 | 37.4 | 26.4 | 48.1 | 22.7 | 24 | 29.6 | 42.1 | 52.1 | 28.1 | 41.4 |
| 161625_r_at | 32 | 42.7 | 59 | 38 | 34.4 | 35.7 | 100.6 | 39.7 | 41.7 | 49.3 | 107.8 | 109.3 | 32 | 47.7 |
| 161626_f_at | 92.9 | 143.9 | 179.2 | 71.3 | 85.1 | 123.5 | 132.7 | 105.1 | 107.4 | 100.2 | 124.4 | 146 | 128.9 | 139.7 |
| 161627_r_at | 138.9 | 202.3 | 174.2 | 204.1 | 191.6 | 153.7 | 264.9 | 172.2 | 136.1 | 182.6 | 175 | 237.7 | 141.6 | 165.1 |
| 161628_r_at | 13.2 | 14.5 | 39.3 | 16.8 | 13.3 | 18.2 | 24 | 14.7 | 10.4 | 25.5 | 20.5 | 27.3 | 19.7 | 31.7 |
| 161629_i_at | 64.6 | 76.9 | 67.4 | 74.7 | 64.7 | 90.2 | 84.7 | 70.3 | 59.6 | 49.5 | 74.2 | 67.5 | 80.8 | 80.4 |
| 161630_i_at | 68.9 | 89.1 | 43.1 | 45.5 | 35.9 | 51.9 | 45.9 | 33.8 | 55.1 | 65.6 | 86.5 | 82 | 63.9 | 76.1 |
| 161631_f_at | 195.1 | 186 | 191.4 | 206.2 | 206 | 137.3 | 182.4 | 209.4 | 281.9 | 235.4 | 172.6 | 158.3 | 134.4 | 224.5 |
| 161632_r_at | 544.9 | 760.4 | 450.9 | 552.7 | 312.8 | 711.6 | 908.3 | 634.9 | 643.2 | 494.9 | 919.7 | 892.9 | 709.8 | 664.7 |
| 161633_r_at | 322.8 | 426 | 392 | 340.1 | 287.9 | 382.6 | 468.9 | 380.1 | 232.5 | 289.1 | 296.6 | 359.4 | 330.7 | 335.6 |
| 161634_r_at | 6.6 | 9.5 | 7.4 | 15 | 4.2 | 3 | 8.9 | 7.2 | 5.4 | 4.1 | 10.1 | 8.7 | 2.6 | 8.8 |
| 161635_f_at | 25.8 | 44.5 | 34.9 | 40.2 | 27.2 | 37.8 | 22.8 | 30 | 22.2 | 27.3 | 44.7 | 45.4 | 31.5 | 19.9 |
| 161636_r_at | 3116.4 | 3712.5 | 4212.2 | 3336.1 | 3676.4 | 3159.2 | 4125.5 | 3987 | 4112.9 | 3343.1 | 3733.9 | 3574 | 4161.9 | 4239.9 |
| 161637_f_at | 24.3 | 10.1 | 14.1 | 13.4 | 17 | 25.7 | 12.6 | 154.1 | 190.2 | 168 | 179.2 | 144.5 | 18 | 22.5 |
| 161638_f_at | 279.2 | 282.1 | 249.2 | 244.5 | 232.9 | 251 | 299 | 232.8 | 253 | 237.2 | 264.4 | 222.7 | 148.9 | 199.3 |
| 161639_f_at | 32.6 | 31.7 | 7.8 | 39.2 | 53.4 | 49.8 | 23.5 | 49.1 | 20.9 | 36.7 | 24.2 | 43.6 | 38.8 | 67.5 |
| 161640_at | 80.2 | 25 | 38.2 | 37.7 | 23.5 | 33.7 | 94.8 | 33.1 | 89.1 | 74.6 | 77.8 | 149.1 | 91.6 | 109.2 |
| 161641_at | 157.2 | 258 | 185.9 | 182.6 | 148.9 | 201.1 | 220.6 | 294.2 | 227.1 | 175.4 | 244 | 241.1 | 191.8 | 202.8 |
| 161642_f_at | 1.5 | 6.2 | 2.3 | 2 | 3.7 | 2.4 | 3.8 | 3.2 | 2.5 | 1.8 | 4.6 | 4.3 | 2.4 | 1.9 |
| 161643_i_at | 13 | 12.4 | 23.1 | 15.2 | 17.4 | 13.1 | 9.2 | 18.8 | 7.4 | 12.2 | 14.6 | 19.7 | 14.1 | 12.8 |
| 161644_f_at | 563.9 | 674.5 | 700.9 | 612.6 | 533 | 611.2 | 723.1 | 670.4 | 545.2 | 562.7 | 590.5 | 547.9 | 572.9 | 657.8 |
| 161645_r_at | 7 | 8.2 | 8.8 | 6.2 | 6.2 | 9.2 | 8.1 | 7.1 | 6.4 | 5.4 | 8.1 | 10.4 | 7 | 8.2 |
| 161646_r_at | 1.9 | 6.8 | 0.4 | 7.1 | 3.8 | 1.1 | 5.8 | 6.5 | 3 | 0.6 | 8 | 0.9 | 0.5 | 1.4 |
| 161647_f_at | 9.8 | 28.3 | 16.2 | 10.8 | 19.7 | 24 | 10.7 | 11.5 | 35.1 | 31.4 | 16.6 | 14.5 | 9.2 | 14.3 |
| 161648_at | 876.9 | 770.8 | 725.8 | 695.5 | 843 | 762.5 | 538.3 | 645.9 | 654.3 | 582.1 | 842.3 | 751.2 | 820.4 | 726.1 |
| 161649_f_at | 6 | 2.9 | 4.2 | 5.9 | 3.7 | 2.9 | 3.3 | 4.6 | 2.1 | 3.2 | 3.3 | 4 | 1.5 | 3.1 |
| 161650_at | 510.7 | 637.5 | 505.6 | 463.3 | 462.8 | 554.3 | 595.3 | 538.4 | 575.1 | 466 | 561.2 | 560.9 | 440.9 | 583.8 |

[illegible]

Moggs et al. Phenotypic anchoring of gene expression changes during estrogen-induced uterine growth

[illegible]

Supplementary Table 1

# Moggs et al. Phenotypic anchoring of gene expression changes during estrogen-induced uterine growth

[illegible]

Supplementary Table 1
